# Genetic basis of resistance to southern corn leaf blight in the maize multi‐parent population and diversity panel

**DOI:** 10.1111/pbi.13967

**Published:** 2023-01-09

**Authors:** Gengshen Chen, Yingjie Xiao, Sha Dai, Zhikang Dai, Xiaoming Wang, Bailin Li, Jennifer S. Jaqueth, Wenqiang Li, Zhibing Lai, Junqiang Ding, Jianbing Yan

**Affiliations:** ^1^ National Key Laboratory of Crop Genetic Improvement Huazhong Agricultural University Wuhan China; ^2^ Hubei Hongshan Laboratory Wuhan China; ^3^ Institute of Crop Science Chinese Academy of Agricultural Sciences Beijing China; ^4^ Corteva Agriscience Johnston IA USA; ^5^ College of Agronomy Henan Agricultural University Zhengzhou China; ^6^ The State Key Laboratory of Wheat and Maize Crop Science and Center for Crop Genome Engineering Henan Agricultural University Zhengzhou China

**Keywords:** southern corn leaf blight, maize disease, joint linkage mapping, genome‐wide association study, resistance loci

## Abstract

Southern corn leaf blight (SLB), caused by the necrotrophic pathogen *Cochliobolus heterostrophus*, is one of the maize foliar diseases and poses a great threat to corn production around the world. Identification of genetic variations underlying resistance to SLB is of paramount importance to maize yield and quality. Here, we used a random‐open‐parent association mapping population containing eight recombinant inbred line populations and one association mapping panel consisting of 513 diversity maize inbred lines with high‐density genetic markers to dissect the genetic basis of SLB resistance. Overall, 109 quantitative trait loci (QTLs) with predominantly small or moderate additive effects, and little epistatic effects were identified. We found 35 (32.1%) novel loci in comparison with the reported QTLs. We revealed that resistant alleles were significantly enriched in tropical accessions and the frequency of about half of resistant alleles decreased during the adaptation process owing to the selection of agronomic traits. A large number of annotated genes located in the SLB‐resistant QTLs were shown to be involved in plant defence pathways. Integrating genome‐wide association study, transcriptomic profiling, resequencing and gene editing, we identified *ZmFUT1* and *MYBR92* as the putative genes responsible for the major QTLs for resistance to *C. heterostrophus*. Our results present a comprehensive insight into the genetic basis of SLB resistance and provide resistant loci or genes as direct targets for crop genetic improvement.

## Introduction

The necrotrophic fungus *Cochliobolus heterostrophus* infects maize leaves, sheaths and ear husks, forming southern corn leaf blight (SLB) symptoms of elliptical to irregular shape and marginal brown necrotic lesions at infected sites. SLB had widespread outbreaks in tropical and subtropical regions, such as the south‐eastern United States, Latin America, Southern Europe and the Yellow‐Huai‐Hai River plain of China, which caused massive losses of maize yield (Wang *et al*., 2014). During the 1970 SLB epidemic, the entire United States maize yield was reduced by an estimated 20%–30% only due to SLB, causing about 1 billion dollars in losses (Ullstrup, 1972). Since the deployment of race T‐resistant and normal cytoplasm maize cultivars after the 1970s, the occurrence of SLB was effectively controlled. However, it is still an important foliar disease, currently predominantly caused by race O, potentially threatening maize production worldwide (Balint‐Kurti *et al*., 2007; Wang *et al*., 2014).

Many quantitative trait loci (QTL) mapping studies have identified the SLB resistance loci in biparental segregating populations. More than 140 QTLs distributed on the ten maize chromosomes were detected, and an obvious enrichment was observed on chromosome 3 with more than 25 loci (Ali *et al*., 2013), of which one major QTL on bin3.04 was identified by several different genetic backgrounds (Balint‐Kurti *et al*., 2006, 2007, 2008; Balint‐Kurti and Carson, 2006; Carson *et al*., 2004; Jiang *et al*., 1999; Negeri *et al*., 2011). One recessive, large‐effect gene *rhm1* located in bin6.01 was another hotspot‐resistant QTL for race O of *C. heterostrophus* (Balint‐Kurti *et al*., 2007; Zaitlin *et al*., 1993). The resistant alleles of two QTLs (bin3.04 and bin6.01) caused moderate yield loss in the absence of significant levels of SLB (Santa‐Cruz *et al*., 2014). For other QTLs, very little is known about how much those disease QTLs contribute to differences in agronomic traits (Frey *et al*., 2011; Santa‐Cruz *et al*., 2014). Despite a large number of QTLs that have been identified, only two of them had been cloned, *rhm1* and *qMdr9.02*. Through fine mapping, the *rhm1* locus was delimited to an 8.56 Kb interval, which contained only one putative gene encoding a lysine histidine transporter1 (LHT1) protein (Zhao *et al*., 2012). One multiple disease resistance (MDR) locus *qMdr9.02* associated with resistance to SLB, northern corn leaf blight (NCLB) and grey leaf spot (GLS) was map‐based cloned (Yang *et al*., 2017). *ZmCCoAOMT2* is the gene underlying the resistance effect at *qMdr9.02*, which encodes a caffeoyl‐CoA O‐methyltransferase involved in the phenylpropanoid pathway and in lignin production (Yang *et al*., 2017). Owing to few cloned genes and little known about the network between SLB resistance and agronomic traits, thus, cloning key resistant genes and investigating the effects of resistant loci that influenced agronomic traits will facilitate accurate maize breeding.

In the last decade, several studies employed genome‐wide association study (GWAS) in the maize nested association mapping (NAM) population and natural diversity panel to dissect the genetic basis of SLB resistance (Kump *et al*., 2011; Wisser *et al*., 2011). The results demonstrated that the genetic basis of SLB resistance is dominated by small additive effects with little epistasis. Many QTLs with small effects were identified in three multi‐parent populations via joint linkage analysis (Lennon *et al*., 2017; Lopez‐Zuniga *et al*., 2019; Negeri *et al*., 2011), and however, represented a small proportion of genetic diversity in maize. In recent years, the multi‐parent design named random‐open‐parent association mapping (ROAM) population was developed, which contained a set of recombinant inbred lines (RILs) families derived from crosses among multiple randomly inter‐crossed founder parents. Those founder lines as elite maize inbred lines had been widely used in maize breeding for the past few decades. The ROAM population had demonstrated its mapping resolution and statistical power for identifying variants of minor effect and low frequency (Liu *et al*., 2017b; Pan *et al*., 2017; Xiao *et al*., 2016), and uncovered the genetic architecture of complex traits, like maize ear traits, kernel size and weight, plant architecture and kernel starch content (Hu *et al*., 2021; Liu *et al*., 2017b; Pan *et al*., 2017; Xiao *et al*., 2016).

In this study, we used ROAM population containing eight RIL families and an association mapping panel (AMP) consisting of 513 diverse maize inbred lines to dissect the genetic basis of SLB resistance. Many QTLs with small‐ and moderate‐additive effects were detected, and most of the putative genes were found to participate in metabolic processes or plant stimulus responses which were highly related to plant immunity. We analysed the resistant allele frequency of those loci in tropical and temperate sub‐populations and found susceptible alleles might be mildly selected during the tropical‐temperate adaptation due to their pleiotropic to agronomic traits. Through GWAS, transcriptomic profiling, resequencing and gene editing, we identified *ZmFUT1* and *MYBR92* as the causal genes responsible for the major QTLs for resistance to *C. heterostrophus*. Our results presented comprehensive insights into the genetic basis of SLB resistance and provided valuable key genes for maize resistance breeding.

## Results

### Phenotypic variation of SLB score index in the ROAM population and AMP population

**Figure 1:**
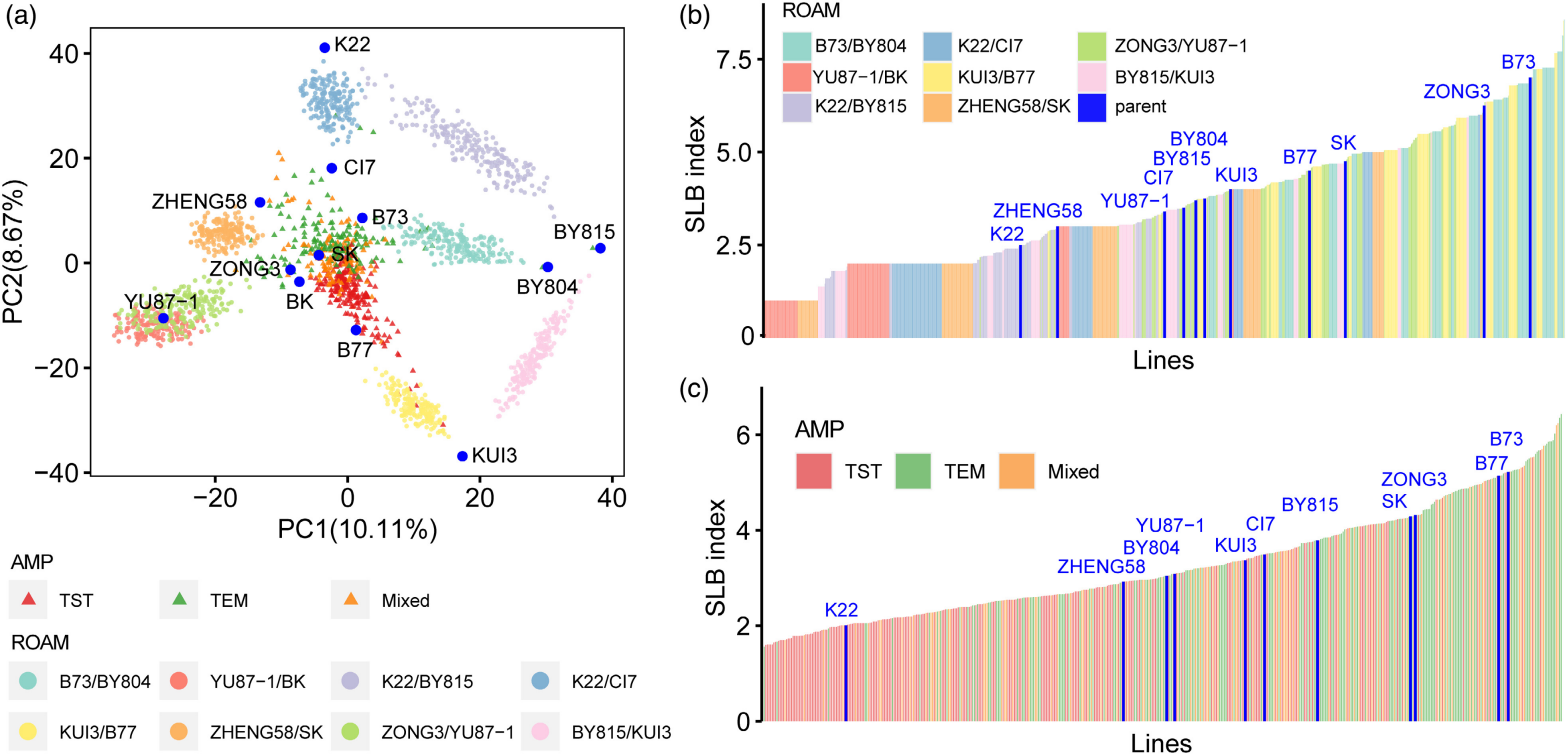
The phenotypic distribution of resistance to southern corn leaf blight in ROAM and AMP populations. (a) Genetic variation among ROAM and AMP populations visualized using a principal component analysis. The founder lines were showed with blue points. TST, tropical lines. TEM, temperate lines. (b) SLB index presented as best linear unbiased predictor (BLUP) scores in ROAM RIL families, except for three RIL families (YU87‐1/BK, K22/CI7, ZHENG58/SK). (c) SLB index in AMP. For ‘a’, ‘b’, ‘c’ plots, each colour represents each RIL family of ROAM or subpopulation of AMP population. For ‘b’ and ‘c’ plots, the founder lines were showed with blue colours by using phenotype of corresponding populations.

The ROAM population, consisting of 1540 lines from eight RIL families (B73/BY804, BY815/KUI3, K22/BY815, K22/CI7, KUI3/B77, YU87‐1/BK, ZHENG58/SK, ZONG3/YU87‐1) derived from 12 founder lines, displayed a wide genetic variation (Figure 1a) and varying resistance to SLB (Figure 1b). The most resistant parental line was K22, and in contrast, B73 was a highly susceptible line, of which the difference was about 4.5 disease scores (Figure 1b). The SLB mean index of eight RIL families also showed significant differences, ranging from 1.81 to 5.83 (lower values with higher resistance; Table S1). The phenotypic distributions of RIL families except for the K22/CI7 family were approximately normal (Figure S1). The heritability ranged from 0.82 to 0.93 with an average of 0.86 (Table S1).

We also investigated the SLB resistance in the AMP population. The SLB indexes ranged from 1.12 to 5.62 and were visually distinguishable (Figure 1c; Figure S2). SLB indexes were significantly correlated among environments with correlation coefficients ranging from 0.47 to 0.94 (Table S2) and SLB resistance had high heritability of 0.89, which suggested that resistance to SLB was mainly controlled by genetic factors. It's worth noting that the tropical maize lines were found to have significantly higher SLB resistance than the temperate lines (*n* = 225/173, *P* = 2.48 × 10^−21^) (Figure S3a). There were 9.55% (49/513) of inbred lines belonged to high resistance varieties (SLB index ≤2), and 86% of those resistance varieties were tropical lines (Figure S3b), which suggested that the tropical sub‐population is a rich source of SLB resistance.

### Identification of the SLB resistance QTLs using multiple populations and analytic approaches

**Figure 2:**
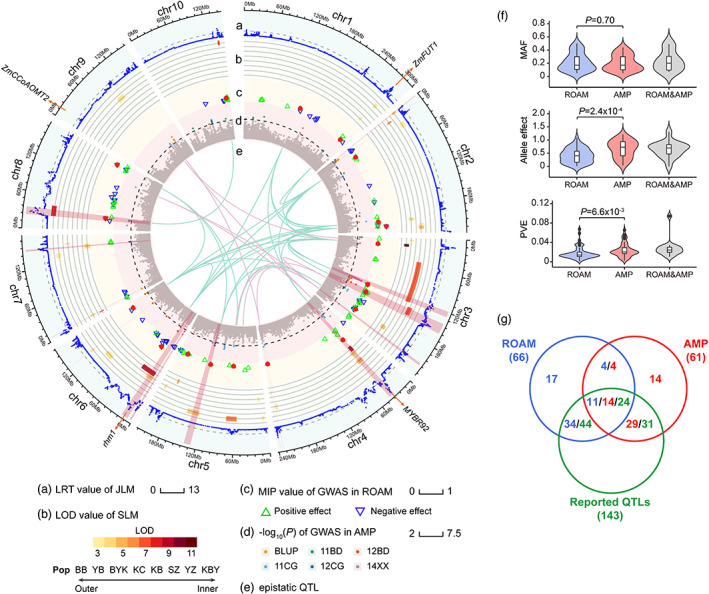
Overview of QTLs for SLB resistance in ROAM and AMP populations. (a) Plot displays the likelihood ratio test (LRT) scores of genome‐wide bins with JLM method. The dashed horizontal line depicts the threshold (LRT = 2.7) (b) SLM QTLs across eight RIL families. The coloured rectangles indicate QTL region and colour density is in the proportion to the logarithm of the odds (LOD) values. (c) Significant SNPs identified by GWAS in ROAM. Blue downward triangles: minor allele decreases SLB index relative to the major allele. Green upward triangles: minor allele increases SLB index relative to the major allele. Red dots: candidate SNPs identified by the final backward model. MIP, model including probability. (d) Manhattan plot for SLB index in five environments and BLUP in the AMP population. The dashed horizontal line depicts the Bonferroni‐adjusted significance threshold (*P* = 1.0 × 10^−5^). The significant SNPs above dashed line are labelled as coloured dots. Orange: BLUP. Light blue: 11CG. Green: 11BD. Navy blue: 12CG. Orange red: 12BD. Purple: 14XX. (e) Significant pair‐wise epistatic interactions in SLB resistance variations. Each line links an epistatic pair of loci. The cyan and pink coloured lines strand for the ROAM and AMP populations respectively. 15 QTLs, both detected in ROAM and AMP populations, were highlight with light red. (f) Comparisons of minor allele frequency (MAF), allele effect and variance explained (PVE) of QTLs among ROAM and AMP populations. (g) The co‐localization among ROAM, AMP and the reported QTLs. The coloured numbers stand for different results, sky blue for ROAM QTLs, red for AMP QTLs, green for reported QTLs.

We used three methods, separate linkage mapping (SLM), joint linkage mapping (JLM) and GWAS in ROAM which had been described in a previous study (Xiao *et al*., 2016), to dissect the architecture of SLB resistance. First, we identified 36 SLM QTLs (LOD ≥ 3), of which nine major QTLs with more than 10% phenotypic variance explained (PVE). A total of 29 QTLs were only identified in one family, while seven QTLs were co‐located among two or three RIL families and were resolved into three consensus QTLs (Figure 2; Table S3). Two consensus QTLs were major QTLs, which possessed 19.53% PVE in BY815/KUI3 and 14.67% PVE in ZHENG58/SK. In summary, those 36 QTLs were summarized into 32 unique QTLs (Table S4).

Joint linkage mapping analysis based on an integrated genetic map of 14 613 bins (a minimal genomic region without recombination) revealed 43 JLM QTLs (LRT ≥ 2.76) on ten chromosomes contributing to SLB resistance, ranging between seven QTLs on chromosome 6 and two QTLs on chromosome 10 (Figure 2a; Table S5). No pair‐wise interaction between QTLs with additive effects was detected. The average length of the QTL interval was 4.1 Mb ranging from 80.9 Kb to 17.1 Mb. About 39.5% (17/43) of JLM QTLs had less than a 1‐Mb interval, in contrast, only 5.6% (2/36) of SLM QTLs had less than a 1‐Mb interval (Figure S4), suggesting that combining multiple genetic populations with JLM analysis resulted in higher mapping resolution than that of SLM.

We next performed GWAS in the ROAM population by using stepwise regression and a resample analysis. A total of 19 SNPs were significantly associated with SLB resistance (Figure 2c; Tables S6 and S7), and jointly explained 51.5% of the total phenotypic variation. One GWAS loci, tagged SNP chr4.S_34473762, was overlapping with SLM QTLs identified by three RIL families (KUI3/B77, YU87‐1/ZONG3 and BY815/KUI3) and JLM QTL 4_34.73. Thirty‐one significant pair‐wise epistatic interactions among the 19 SNPs were detected at *P* < 2.92 × 10^−4^ (0.05/*N*, *N* is 171 pairwise epistatic interactions among 19 significant SNPs), with small PVE values ranging from 0.93% to 6.13% (Figure 2e; Table S8). The epistatic effects explained only a small additional PVE (6.48%) beyond the additive model, further confirming epistatic effects are less important for SLB resistance as compared to QTL main effects. Those ROAM QTLs (32 SLM QTLs, 43 JLM QTLs and 19 GWAS loci) were integrated into 66 QTLs, of which about one‐third (20/66) was supported by two or three analytic methods (Figure S5). Those results indicated that multiple statistical methods are complementary for fully identifying genomic loci underlying SLB resistance.

We further performed GWAS in the AMP population by using 1.25 M SNPs (MAF ≥ 0.05), and detected 337 SNPs significantly associated with SLB resistance (*P* ≤ 1 × 10^−5^; Figure 2d; Figure S6). Those significant SNPs were divided into 61 genetic loci (Tables S9 and S10), which explained 74.5% of the total phenotypic variation. Although the epistatic interactions among those loci were detected at *P* < 2.73 × 10^−5^ (0.05/*N*, *N* is 1830 pairwise epistatic interactions; Figure 2e; Table S11), those epistatic effects totally explained only 9.88% of phenotypic variation, suggesting again that SLB resistance was predominantly controlled by QTL main effects.

Among those QTLs, 15 ROAM QTLs were overlapped the 18 GWAS loci from the AMP population, and those QTLs/loci were integrated into 109 unique QTLs for SLB resistance. It's noted that 51 ROAM QTLs were specifically identified in ROAM but not in the AMP population, possibly because those ROAM‐specific QTLs contributed less variance in the AMP population due to the lower allele effects, compared to those AMP‐specific QTLs (effects: *P* = 2.4 × 10^−4^; PVE: *P* = 6.6 × 10^−3^; Figure 2f). It suggested that the multi‐parent population is complementary for mining genetic loci underlying SLB resistance to the diverse inbred panel. We observed a high proportion (74/109) of resistant QTLs were co‐located with previously reported QTLs (Figure 2g; Figure S7; Table S12; Balint‐Kurti *et al*., 2006, 2007; Balint‐Kurti and Carson, 2006; Carson *et al*., 2004; Kump *et al*., 2011; Lennon *et al*., 2017; Liu *et al*., 2011; Lopez‐Zuniga *et al*., 2019; Negeri *et al*., 2011; Zwonitzer *et al*., 2009). Though, our study identified 35 novel QTLs beneficial for further understanding maize SLB natural resistance variation, containing 21 ROAM QTLs and 18 AMP QTLs. Among them, four novel loci were conservatively detected in two populations, contributing potentially to the SLB resistance with decreasing 0.485 SLB index in average.

### The genetic architecture of SLB resistance in maize diverse inbred lines

**Figure 3:**
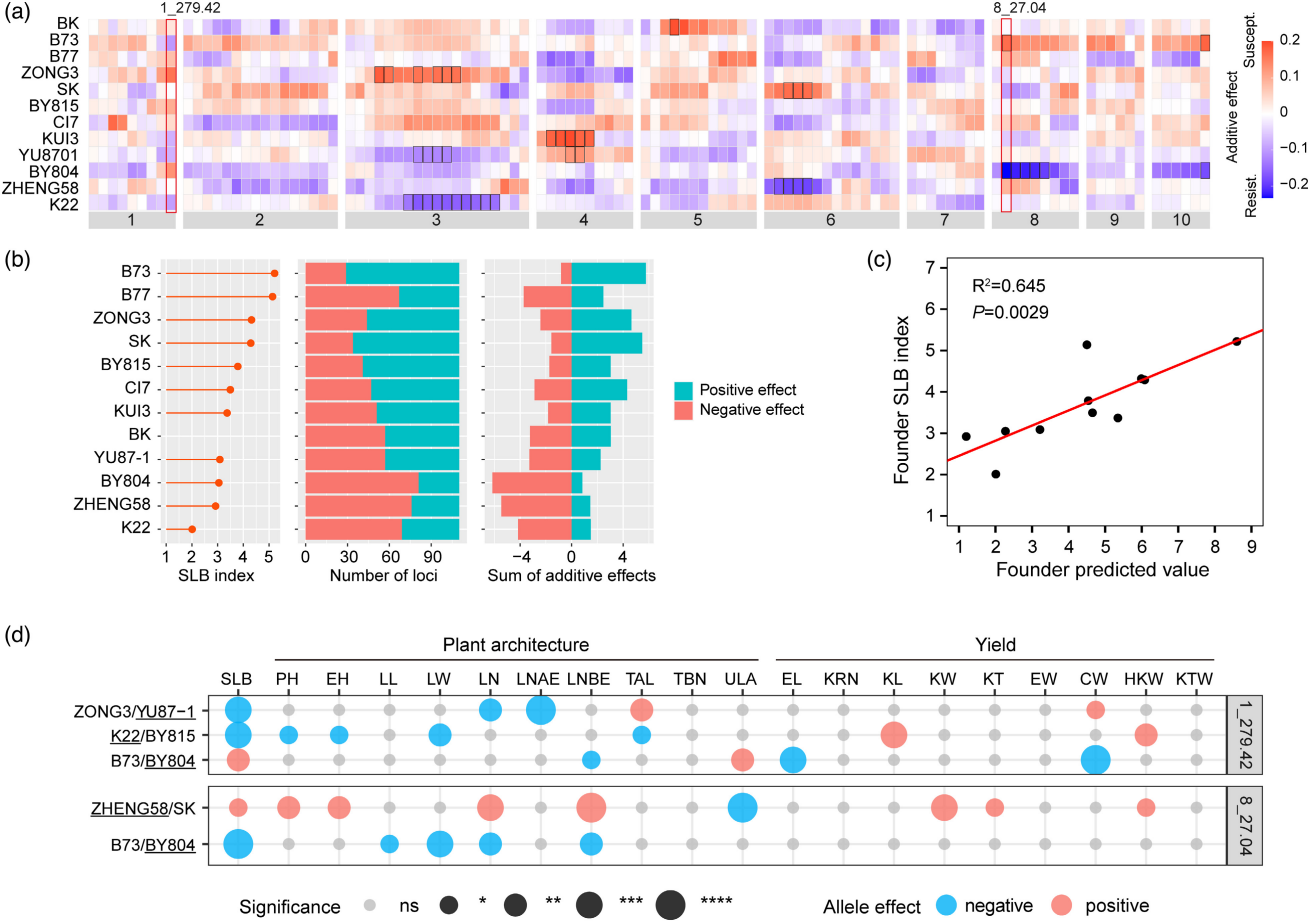
The genetic attributes of SLB diversity in 12 ROAM parental lines. (a) The additive effects of 12 parental lines at 109 QTLs. Two QTLs, 1_279.42 and 8_27.04, were highlighted with red rectangles. The allelic effect size represents each parental allelic effect (rows) relative to the mean value. The relative value is coded by colour in the legend. Allelic effects significantly different from mean value at 5% false discovery rate were surrounded by bold lines. (b) The numbers and accumulated additive effects of positive and negative alleles in 12 parental lines. (c) Predicted phenotypes of 12 founder lines based on QTL estimates. (d) The allelic effects of SLB resistance and agronomic traits at QTL 1_279.42 and 8_27.04. Allelic effect of resistant parental lines relative to susceptible parental lines were shown with sky blue (negative) and light red colours (positive). Four resistant parental lines (ZHENG58, BY804, YU87‐1 and K22) were underlined. CW, cob weight; EH, ear height; EL, ear Length; EW, ear weight; HKW, hundred kernel weight; KL, kernel length; KRN, kernel row number; KT, kernel thickness; KTW, kernel test weight; KW, kernel width; LL, ear leaf length; LN, leaf number; LNAE, leaf number above ear; LNBE, leaf number below ear; LW, ear leaf width; PH, plant height; TAL, tassel main axis length; TBN, tassel branch number; ULA, upper ear leaf angle. The differences were analysed by Student's two‐sided *t*‐test, *P < 0.05; **P < 0.01; ***P < 0.001; ****P < 0.001.

To better understand the genetic mechanism of SLB resistance in 12 parental lines, we analysed our identified 109 QTLs in multiple populations. In ROAM population, the most significant bins with each QTL were selected to evaluate the allelic effects of 12 parental lines. We found that small‐effect alleles underlie all those QTLs for SLB resistance (Figure 3a). The significant allelic effects for parental lines ranged from absolute values 0.12 to 0.23, with an average of 0.17 (Figure S8), and those significant resistant alleles come from four parental lines (K22, ZHENG58, BY804 and YU87‐1). We found that the resistant levels of parental lines were significantly correlated to simply a resistant allelic number (*P* = 3.63 × 10^−7^) and summed resistant effects (*P* = 2.06 × 10^−10^). For example, line K22, the highest SLB resistance, possessed 69 resistant alleles and accumulated 4.16 resistant effects, while the most susceptible line B73 carried 29 resistant alleles and accumulated 0.82 resistant effects (Figure 3b). Overall, the 109 QTLs enabled the explanation of a large portion (64.5%) of SLB resistance in the 11 found lines based on the model built in the ROAM offspring lines (Figure 3c). In combination with plant architecture and yield‐related traits in the ROAM population released previously (Liu *et al*., 2017b; Pan *et al*., 2017; Xiao *et al*., 2016), we found that the 109 SLB QTLs influenced multiple agronomic traits (Table S13). At QTL 1_279.42, the lines carrying the resistant allele (K22, YU87‐1 and B73) showed higher yields (higher cob weight or hundred kernel weight) in K22/BY815, ZONG3/YU87‐1 and B73/BY804 RIL families, respectively (Figure 3d), indicating that such resistant alleles as ideal targets have potential to improving resistance and yields simultaneously. At another major QTL 8_27.04, resistant parental line ZHENG58 contains the susceptible allele, and lines carrying this susceptible allele performed better plant architecture (smaller upper leaf angle) and higher yields (higher hundred kernel weight) in ZHENG58/SK RIL family, while resistant parental line BY804 has resistant allele at this QTL and lines carrying this resistant allele showed a smaller and incompact plant architecture in B73/BY804 RIL family (Figure 3d), suggesting that the resistant allele of major QTL may cause an adverse impact on plant architecture and severe yield losses. Those results further suggested that those elite founder lines own the potential for continual improvement.

**Figure 4:**
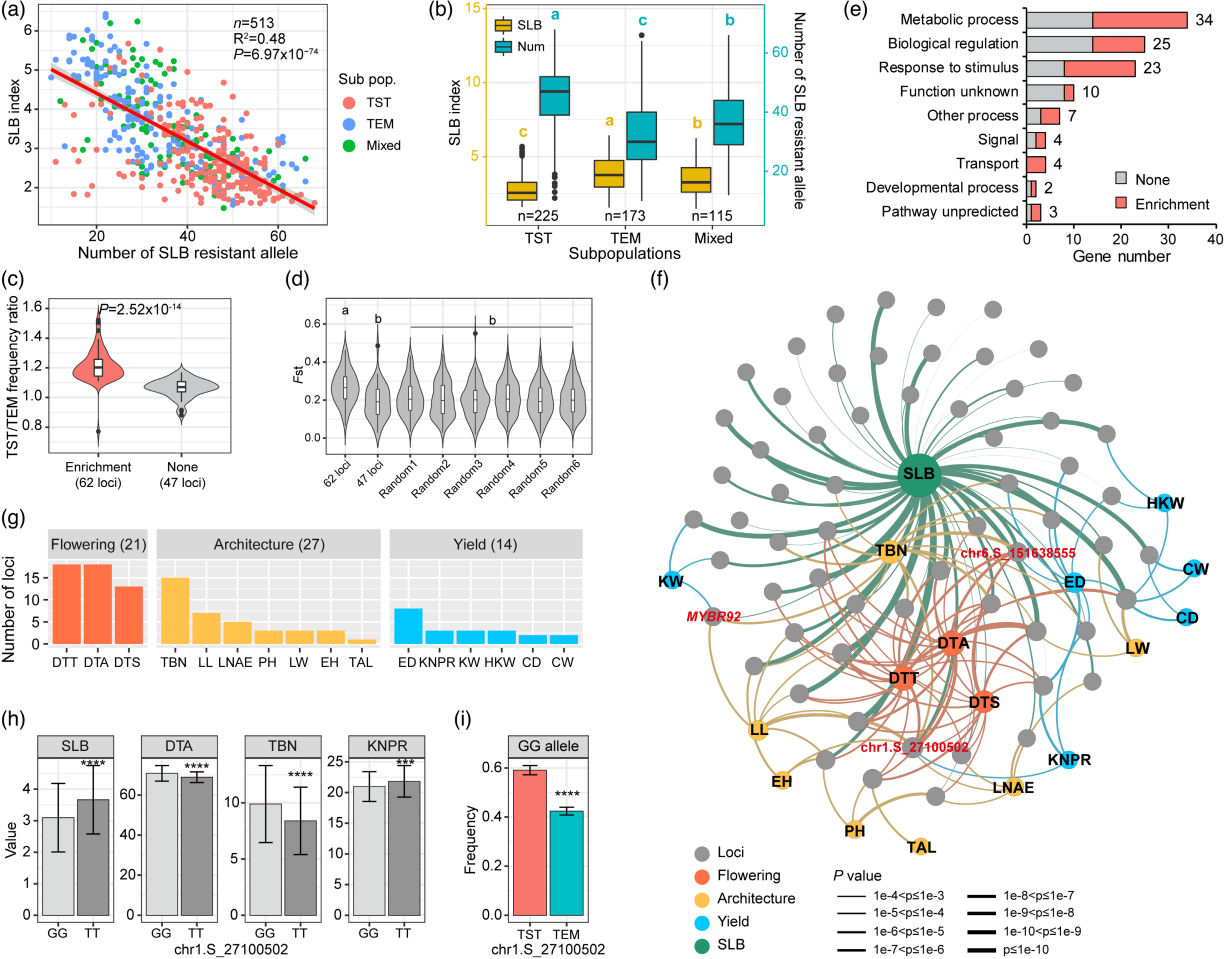
The maize adaptation contributes to the SLB resistance distribution. (a) Plot for the SLB resistance against the accumulation of resistant alleles. The coloured points present inbred lines belonged to corresponding tropical (TST) and temperate (TEM) subpopulations. (b) SLB resistance and resistant allele numbers of inbred lines from TST and TEM subpopulations. Difference letters indicate significant difference at fdr ≤0.05 level via Fisher's LSD test. (c) The frequency ratio of the resistant alleles of 109 loci (62 enrichment loci and 47 non‐enrichment loci) between TST and TEM lines. (d) The fixation index (*F*_ST_) value among 62 loci, 47 loci and random loci. (e) Histogram of biological pathway annotations of candidate genes for 109 loci. The candidate genes for 62 adapted loci were displayed with red colour. (f) Association network among SLB resistance and agronomic traits in AMP. The nodes represent SLB resistance and agronomic traits, and the SLB‐resistant loci. The edges between loci and different traits are linked by the significance. Only 62 adapted loci were displayed. (g) Number of loci linked to agronomic traits. The total number of loci affecting flowering time, plant architecture and yield traits were shown in brackets. (h) The allelic effects of chr1.S_27100502 one SLB resistance and agronomic traits. The differences were analysed by Student's two‐sided *t*‐test, ***P < 0.001; ****P < 0.001. (i) Difference of resistant allele frequency between TST and TEM lines at chr1.S_27100502. CD, cob diameter; CW, cob weight; DTA, days to anthesis; DTS, days to silking; DTT, days to tasselling; ED, ear diameter; EH, ear height; HKW, hundred kernel weight; KNPR, kernel number per row; KW, kernel width; LL, ear leaf length; LNAE, leaf number above ear; LW, ear leaf width; PH, plant height; TAL, tassel main axis length; TBN, tassel branch number.

In a broader diversity of maize inbred lines, we found that the accumulation of SLB‐resistant alleles is capable to explain 48% variance of SLB resistance (Figure 4a), which indicated that the genetic improvement of the SLB resistance can be achieved by linearly stacking beneficial haplotypes. That is, the highly resistant inbred lines (SLB index ≤2) generally carried 15 beneficial alleles more than the remaining ones (Figure S9a). We further found that the tropical maize inbred lines are significantly more resistant to SLB than the temperate inbred lines (*P* = 2.48 × 10^−21^), which may be due to that the tropical lines accumulated more SLB‐resistant alleles than the temperate ones (*P* = 1.04 × 10^−18^) (Figure 4b; Figure S9b). Interestingly, it found that 57% (62/109) QTLs had different resistant allele frequencies between tropical and temperate lines (Figure 4c; Figure S10a; Table S14), and also showed significantly higher fixation index (*F*_ST_) than the whole genome background (*P* = 1.87 × 10^−4^; Figure 4d; Figure S10b). It suggested that those SLB QTLs were probably involved in the maize adaptation process from tropical to temperate regions. The resistant alleles for 62 QTLs were more frequently existed in tropical than temperate lines, except for one locus (peak SNP: chr5.S_101295764; Table S14). Gene ontology analysis for candidate genes underlying 62 resistant QTLs indicated that most of the candidate genes enriched in metabolic process, biological regulation and response to stimulus (Figure 4e). As those pathways were also involved in plant growth and development, we further analysed the influence of those resistant QTLs on agronomic traits. We observed that 37 of 62 QTLs were able to significantly influence two or more of 17 agronomic traits (*P* < 0.001; Figure 4f; Figure S11). There were 21 SLB QTLs that affected the maize flowering time, 27 QTLs affecting plant architecture and 14 QTLs affecting yield traits (Figure 4g), which is relatively higher than that of 47 non‐adapted QTLs (Figure S12). In the absence of a disease outbreak, the haplotypes for high yield may be the only considered status that may explain the outcome of SLB allele frequency after the maize tropical‐temperate adaptation. For example, at the QTL (peak at chr1.S_27100502), the GG allele can significantly enhance the SLB resistance relative to the TT allele (*P* = 1.61 × 10^−7^), but the resistant type (GG) resulted in the delayed flowering time, more tassel branch numbers, and fewer kernel number per row (Figure 4h). For another QTL chr6.S_151638555, similar phenomena were observed that the TT allele can significantly reinforce SLB resistance (*P* = 2.3 × 10^−9^), and meanwhile, delay flowering time, increase tassel branch numbers and lessen ear diameter (Figure S13a). This may be the reason why the resistant allele frequency was significantly reduced in the temperate lines (Figure 4i; Figure S13b). Those results demonstrated that the SLB resistance loci may contribute to the maize adaptation through the pleiotropic mechanisms, and reinvention of the resistant beneficial alleles in the tropical germplasm may be further exploited.

### Identification of *ZmFUT1* and *MYBR92* responsible for SLB resistance

**Figure 5:**
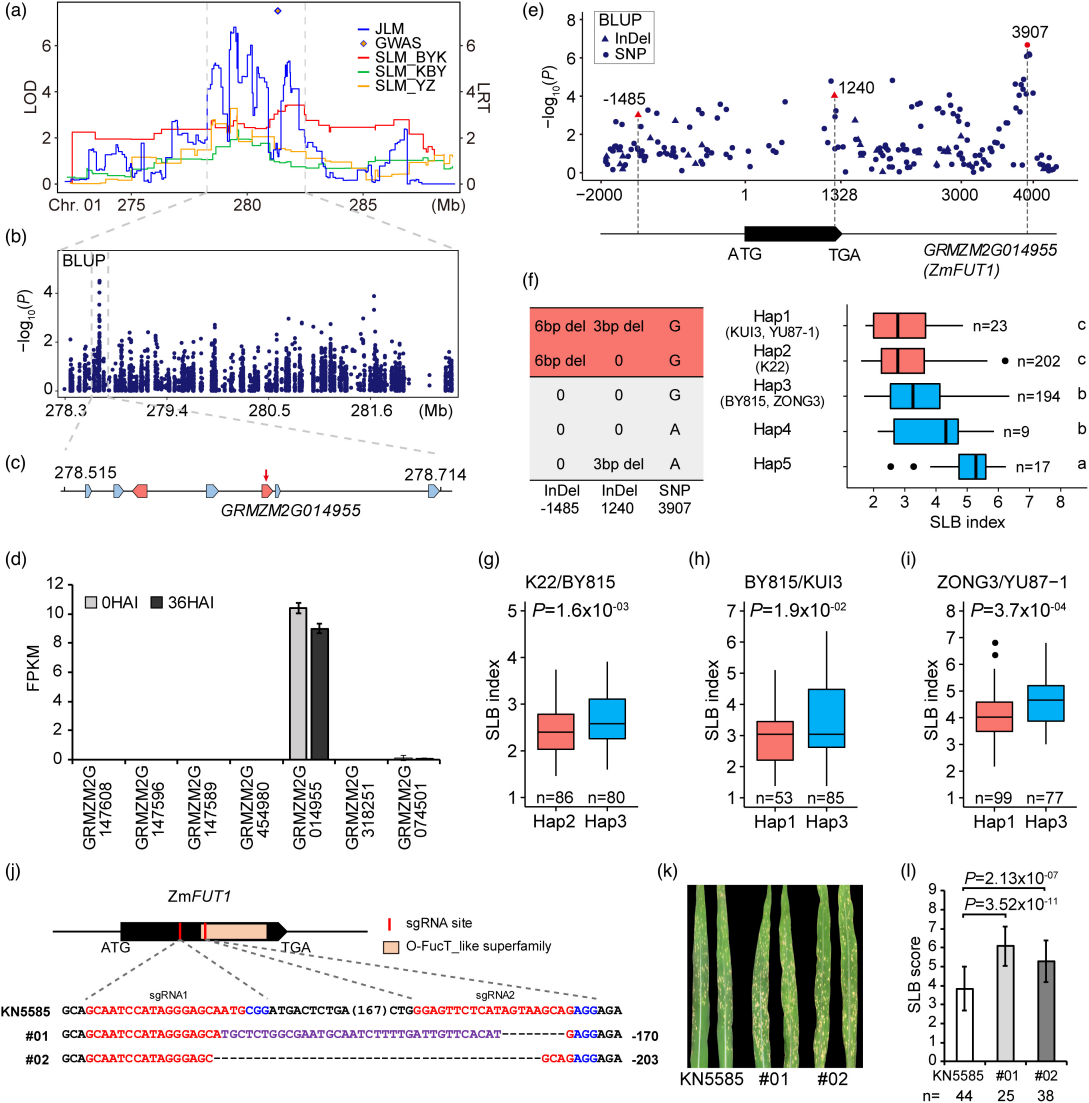
Identification of *ZmFUT1* as the candidate gene for SLB resistance. (a) QTL 1_279.42 was detected via SLM, JLM and GWAS in ROAM population. Red, green and orange lines represent the LOD value of SLM method in three RIL families: K22/BY815 (BYK), BY815/KUI3 (KBY) and ZONG3/YU87‐1 (YZ) respectively. Blue line represents the LRT value of JLM method. A diamond dot represents the significantly associated SNP from GWAS result. (b) Manhattan plot for SLB index in the AMP population at QTL 1_279.42 locus. (c) The annotated genes indicated by blue boxes in a 100‐Kb region on each side of the peak SNP. Red arrow presents peak SNP. The light red boxes present the genes expressed in the leaf tissue according to B73 transcriptomic profiling data. (d) Expression analysis of candidate genes in B73 inoculated with *C. heterostrophus* at two‐time points, 0 HAI and 36 HAI. (e) Local Manhattan plot of gene‐based association mapping. Dots represent SNPs and triangles represents InDels. One SNP 3907, two Indel, −1485 and 1240 were highlighted with red colour. (f) Haplotypes of *ZmFUT1* in AMP population. *n* represents the number of corresponding haplotype group. The boxplot of SLB index for each haplotype is displayed at the right side. Difference letters indicate significant difference at fdr ≤0.05 level via Fisher's LSD test. (g–i) Boxplots for SLB index based on the haplotypes (Hap.) for *ZmFUT1* in three RIL families, K22/BY815 (g), BY815/KUI3 (h) and ZONG3/YU87‐1 (i). (j) CRISPR/Cas9‐induced genome editing of *ZmFUT1* at two sgRNAs sites. Two sgRNAs targeted on the gene coding sequence. For #01 event, 35 bp insertion sequence was marked as purple colour. (k, l) The SLB disease phenotype (k) and SLB scores (l) of KN5585 and *zmfut1*‐knockout lines inoculated with *C. heterostrophus* pathogen. Values are means ± SD. For figure ‘d’, ‘g’, ‘h’, ‘i’, ‘l’, differences between groups or haps were analysed by Student's *t‐*test.

To gain further insights into SLB resistance, we attempted to identify the causal genes of major SLB‐resistant QTLs. We found a JLM QTL (278.27–281.93 Mb) on chromosome 1 was co‐located with SLM QTLs in K22/BY815 (279.12–283.55 Mb), ZONG3/YU87‐1 (277.85–280.21 Mb) and BY815/KUI3 (278.5–280.2 Mb) RIL families. One significant SNP at 281.32 Mb was identified via GWAS within this QTL region in the ROAM population (Figure 5a). We further found a significant signal (MLM, chr1.S_278667428, *P* = 3.1 × 10^−5^) at this QTL region in the AMP population (Figure 5b). In the 100‐Kb flanking region of peak SNP chr1.S_278667428, seven genes were annotated (Figure 5c). We also found that only gene *GRMZM2G014955* was expressed at 36 h after inoculation (HAI), whereas the others were not expressed (Figure 5d). Gene *GRMZM2G014955* encodes protein O‐fucosyl transferase 1 (ZmFUT1) that transfers O‐fucose from GDP‐fucose to serine/threonine residue of proteins. In a previous study, several fucosyl transferases mutants, *spy*, *fucTa fucTb* and *fut4 fut6* exhibited compromised plant defence including apoplastic, stomatal defences, PTI and ETI in *Arabidopsis*, suggesting that protein fucosylation was involved in plant immunity (Zhang *et al*., 2019). Thus, we selected *ZmFUT1* as the most likely candidate gene for this QTL.

Through resequencing of this gene, we detected a much more significant signal with SNP 3907 (*P* = 2.15 × 10^−7^, MLM, *n* = 507) at downstream of this gene. In the AMP population, three GWAS significant variants (two indels −1485, 1240 and one SNP 3907) categorized this gene into five haplotypes (Figure 5e,f). The RIL founder lines KUI3 and YU87‐1 belonged to haplotype 1, while another founder line K22 belonged to haplotype 2. The susceptible founder lines BY815 and ZONG3 belonged to haplotype 3. Lines with haplotype 1 or 2 had much more significant resistance than those with haplotype 3 in the AMP population (Hap1/Hap3: *P* = 0.048, *n* = 23/194; Hap2/Hap3: *P* = 4.32 × 10^−5^, *n* = 202/194; Figure 5f). The similar results were found in RIL families (Hap1/Hap3, *P* = 0.019, *n* = 53/85 for BY815/KUI3 RIL; *P* = 3.7 × 10^−4^, *n* = 99/77 for ZONG3/YU87‐1 RIL; Hap2/Hap3, *P* = 0.0016, *n* = 86/80 for K22/BY815 RIL; Figure 5g–i). To test the relationship between *ZmFUT1* expression and SLB resistance, we analysed the gene expression level derived from RNA‐seq data for leaf tissues and found that the expression level of *ZmFUT1* was marginally correlated with SLB resistance, while the expression level had no significant difference between 0 HAI and 36 HAI (Figure S14). Thus, it suggested that function of *ZmFUT1* on SLB resistance may not lie on the transcriptional level.

We further obtained two independent *zmfut1*‐knockout lines via CRISPR/Cas9 genome‐editing system in maize inbred line KN5585. One line contains 170 bp deletion and another line contains 203 bp deletion in the coding sequence, which both caused a loss of function (Figure 5j). At 3 days after inoculation with *C. heterostrophus* pathogen, the knockout lines had significantly lower SLB resistance (higher scores), much more necrotic lesions and fungal biomass than the wild‐type plants (wt/#01: *P* = 3.52 × 10^−11^, *n* = 44/25; wt/#02: *P* = 2.13 × 10^−7^, *n* = 44/38) (Figure 5k,l; Figure S15). We collected the agronomic traits of those *zmfut1* mutants and found that flowering time, plant architecture and yield‐related traits were not significantly different between wild‐type lines and mutants (Figure S16). Hence, those results suggested that *ZmFUT1* is the gene underlying the resistant effect at this QTL.

**Figure 6:**
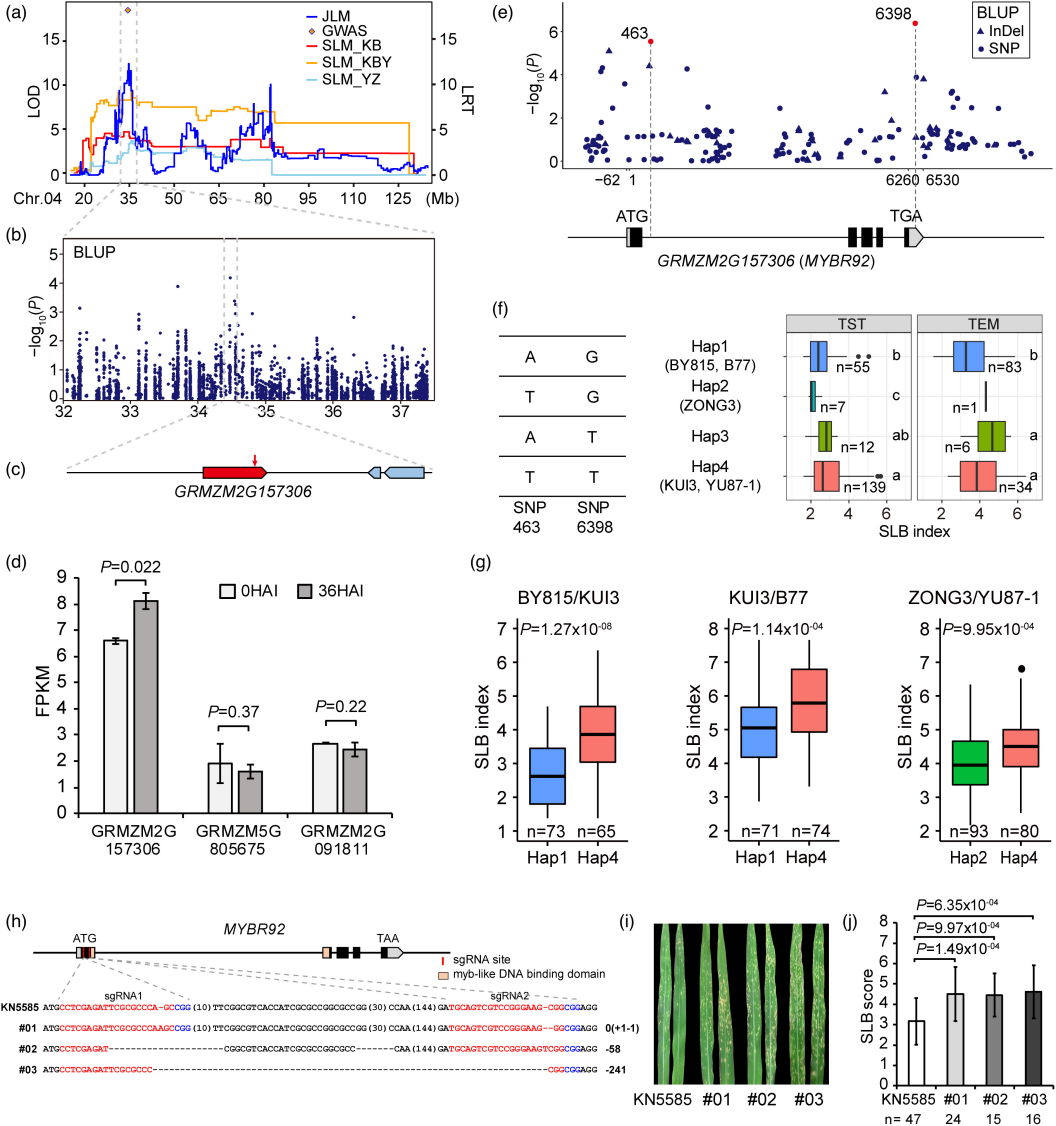
Identification and functional verification of *MYBR92* for SLB resistance in maize. (a) QTL 4_34.73 was detected via SLM, JLM and GWAS in the ROAM population. (b) Manhattan plot for SLB index in AMP at this QTL region. (c) The annotated genes indicated by blue boxes in a 100‐Kb region on each side of the peak SNP. Red arrow presents peak SNP. The light red boxes present the genes expressed in the leaf based on B73 transcriptomic profiling. (d) Expression analysis of candidate genes in B73 line inoculated with *C. heterostrophus* at two‐time points, 0 and 36 h (0 and 36 HAI). (e) Local Manhattan plot of gene‐based association mapping. Dots represent SNPs and triangles represents InDels. SNP 463 and SNP 6398 were highlighted with red colour. (f) Haplotypes of *MYBR92* in the AMP population. The SLB index distribution for each haplotype is displayed on the right side. Difference letters indicate significant difference at fdr ≤0.05 level via Fisher's LSD test. (g) Boxplots for SLB index based on the haplotypes (Hap.) for *MYBR92* in three RIL families, BY815/KUI3 (KBY), KUI3/B77 (KB) and ZONG3/YU87‐1 (YZ). (h) CRISPR/Cas9‐induced genome editing of *MYBR92* at two sgRNAs sites. Two sgRNAs targeted on the coding sequence of gene *MYBR92* exon1. (i, j) The SLB disease phenotype (i) and SLB score (j) of KN5585 and *mybr92‐*knockout lines inoculated with *C. heterostrophus* pathogen. Values are means ± SD. For plot ‘d’, ‘g’ and ‘j’, differences between haplotypes or groups were analysed by Student's *t*‐test.

Another major QTL on chromosome 4 was found to influence SLB resistance. This QTL was located within 32.06–41.11 Mb using JLM analysis, while simultaneously detected by SLM analysis in three RIL families (KUI3/B77, 32.06–43.06 Mb; ZONG3/YU87‐1, 31.71–42.92 Mb; BY815/KUI3, 31.58–37.44 Mb). GWAS identified one significant SNP at 34.47 Mb within this QTL region in ROAM population (Figure 6a). Meanwhile, we further found a signal (MLM, chr4.S_34474146, *P* = 6.48 × 10^−5^) in this region in the AMP population (Figure 6b). Three genes (*GRMZM2G157306*, *GRMZM5G805675* and *GRMZM2G091811*) were annotated in the 100‐Kb flanking the peak SNP chr4.S_34474146 (Figure 6c). Only *GRMZM2G157306* were expressed significantly differently at 36 HAI compared with 0 HAI (*P* = 0.022), while the remaining two genes did not show differential expression (Figure 6d). *GRMZM2G157306* (*MYBR92*) encodes an MYB‐like transcription factor. In the previous study, the Arabidopsis *Botrytis Susceptible1* (*BOS1*, *AtMYB108*) gene, encoding an MYB transcription factor, was found to be required to restrict the spread of two necrotrophic pathogens (Mengiste *et al*., 2003). Therefore, we selected *MYBR92* as the most likely candidate gene for this QTL.

Through resequencing of *MYBR92*, we found that two variants (SNP 463 and SNP 6398) in the intron and 3′ untranslated regions deciphered four haplotypes in the AMP population (Figure 6e). The RIL founder susceptible lines BY815, B77 and ZONG3 belonged to haplotype 1 and haplotype 2, while resistant lines KUI3 and YU87‐1 belonged to haplotype 4. Statistically, lines with haplotype 1 or 2 had a significantly higher SLB resistance than those with haplotype 4 in the sub‐population of AMP (TST: Hap1/Hap4, *P* = 0.0039, *n* = 55/139; Hap2/Hap4, *P* = 1.01 × 10^−4^, *n* = 7/139; TEM: Hap1/Hap4, *P* = 0.041, *n* = 83/34; Figure 6f). The significant differences in SLB resistance between those haplotypes were also found in BY815/KUI3, KUI3/B77 and ZONG3/YU87‐1 RIL families (*P* = 1.27 × 10^−8^, *n* = 73/65; *P* = 1.14 × 10^−4^, *n* = 71/74; *P* = 9.95 × 10^−4^, *n* = 93/80 respectively; Figure 6g).

To validate this gene function, we generated *mybr92*‐knockout lines using CRISPR/Cas9 technology. All three independent knockout lines contained frameshift mutations, which caused the loss of function (Figure 6h). At 3 days after inoculation with *C. heterostrophus* pathogen, the knockout plants had significantly lower SLB resistance (higher scores), much more necrotic lesions and fungal biomass than the wild‐type plants (wt/#01: *P* = 1.49 × 10^−4^, *n* = 47/24; wt/#02: *P* = 9.97 × 10^−4^, *n* = 47/15; wt/#03: *P* = 6.35 × 10^−4^, *n* = 47/16) (Figure 6i,j; Figure S17). The flowering time, plant architecture and yield traits of *mybr92*‐knockout lines had no significant difference in comparison with wild‐type lines (Figure S18). Those results indicated that *MYBR92* is responsible for this SLB‐resistant QTL on chromosome 4.

To determine the favourable haplotype in two novel putative genes *ZmFUT1* and *MYBR92*, we conducted haplotype‐based association analysis using the five polymorphism sites. One associated haplotype, comprising three polymorphism sites (InDel‐1485 in *ZmFUT1*, SNP 463 and SNP 6398 in *MYBR92*), was found to be significantly different contributions to the SLB resistance with the remaining haplotypes together (*P* = 3.81 × 10^−17^; Figure S19). The combination of resistant alleles for those three sites displayed high SLB resistance (*ZmFUT1a* + *MYBR92a* + *MYBR92b*, the mean of SLB index: 1.98). Those results suggested that this combination of resistant alleles was the favourable haplotype.

## Discussion

A limited study of maize SLB resistance had been reported using different population designs (Kump *et al*., 2011; Lennon *et al*., 2017; Lopez‐Zuniga *et al*., 2019; Negeri *et al*., 2011). The first study used three RIL families with 460 lines derived from crosses B73 × CML254, CML254 × B97, and B97 × Kil4, respectively (Negeri *et al*., 2011). The second study was conducted in the NAM population comprising 5000 lines derived from crosses between B73 and 25 diverse inbred lines (Kump *et al*., 2011). The third study employed 774 BC_4_S_2_ near‐isogenic lines derived from crosses between 10 different teosinte accessions and B73 (Lennon *et al*., 2017). The fourth study used eight BC_3_F_4:5_ CSSL populations containing 1611 lines developed from crosses between four multiple disease‐resistant lines (NC304, NC344, Ki3, NC262) and two multiple disease‐susceptible lines (Oh7B, H100; Lopez‐Zuniga *et al*., 2019). Our study utilized eight RIL families with 1540 lines derived from crosses between 12 elite inbred lines (Pan *et al*., 2016; Xiao *et al*., 2016). In addition, we carried out GWAS in a large population with high‐density SNPs (513 diverse inbred lines, 1.25 M SNPs). These results showed that there are different SLB resistance QTLs in different genetic backgrounds, and the materials in tropical backgrounds often have much higher resistance levels. We presented an explanation that the tropical lines possessed more resistant alleles, and gathered most of the resistant loci in adaptation regions (Figure 4). Thus, the tropical lines and resistant loci under adaptation regions will be the key sources in the future genetic improvement of maize disease resistance. In the maize adaptation process, many traits were undergone selection, like plant architecture, flowering time and biotic stresses, to successfully adapt to temperate climates (Liu *et al*., 2015). In the absence of disease pressure, breeders and farmers preferred to select cultivars with early flowering time and high yield performance, which resulted in the losses of the resistant loci in the meantime according to this study. So well understanding about the relationship between resistant loci and agronomic traits will provide accurate guidance for maize breeding.

In the current study, we identified many QTLs with three methods in the ROAM population. We noticed that one QTL 4_34.73 on chromosome 4 was identified among three RIL families (ZONG3/YU87‐1, BY815/KUI3, KUI3/B77), and the QTL bin 4.05 of the NAM population was also mapped to this region (Kump *et al*., 2011). It is interesting that no overlapping QTL was found in those RIL families with common parents, such as K22/BY815 and K22/CI7, YU87‐1/BK and ZONG3/YU87‐1, inferring the large difference in the genetic background of the RIL families even though they share a common parent. About 56.3% (18/32) and 43.8% (14/32) SLM QTLs were, respectively, overlapped with JLM QTLs and GWAS loci of the ROAM population, and GWAS loci of the AMP population, meanwhile, 71.9% (23/32) SLM QTLs were co‐located with the previous reported QTLs (Table S12), suggesting that most of the resistant QTLs to SLB are reliable and reproducible. Similar results were observed with maize ear and kernel‐related traits (Liu *et al*., 2017b; Xiao *et al*., 2016). In a natural population, GWAS had successfully identified hundreds of common genetic variants associated with complex traits, while JLM and GWAS in the multi‐parent population had the power to identify QTLs of minor effects and low frequency (Xiao *et al*., 2016, 2017), which may be the explanation that 13.7% (15/109) of loci were detected in both genetic populations (Figure 2f,g). Thus, the diverse mapping populations and different statistical methods used in this study can complement each other, providing the best opportunity for us to comprehensively understand the genetic basis of SLB resistance.

Several candidate genes identified by GWAS were involved in plant disease‐resistance pathways. A total of 25 candidate genes were enriched in stimulus‐response, involved in water deprivation, cold stimulus, stress and carbohydrate stimulus, which were associated with pathogen infections (Saijo and Loo, 2019). A total of 37 candidate genes were linked to biological pathways involved in metabolic process, including four genes (*GRMZM5G844894*, *GRMZM2G122277*, *GRMZM2G110145* and *GRMZM2G165530*) related to the xylan biosynthetic process (Table S12). Xylan is one of the important components of the plant cell wall, and its content and degree of acetylation affect plant penetration resistance against pathogens (Li *et al*., 2019; Yang *et al*., 2017). Therefore, our results reinforce prior findings that xylan metabolism plays an important role in resistance to plant diseases such as SLB.

In this study, 25 candidate genes were involved in biological regulation (Figure 4e), and several genes encoded transcription factors, like MYB, zinc fingers, ERF, MADS‐box, WRKY and NAC proteins, which had been uncovered to regulate immune responses when plants were confronted by pathogens (Tsuda and Somssich, 2015; Zhao *et al*., 2022). The MYB transcription factors are key factors in regulating networks of development, metabolism and responses to biotic and abiotic stresses. Several reported MYB proteins have functions in plant immunity, like AtMYB30, AtMYB44, AtMYB51 and AtMYB108 (Dubos *et al*., 2010; Li *et al*., 2016). The *atmyb108* (*bos1*) mutant enhanced the susceptibility to two necrotrophic pathogens (Mengiste *et al*., 2003). AtMYB108 physically interacts with BOI, a RING E3 ligase, and is ubiquitinated by BOI, which contributes to plant defence by suppressing disease‐associated cell death (Luo *et al*., 2010). We observed a similar phenomenon that maize knockout mutants *mybr92* increased susceptibility to necrotrophic pathogen *C. heterostrophus*, inferring a common host response strategy against necrotrophic pathogens. Additional studies will be necessary to explore the underlying molecular mechanism.

Fucosyltransferases (FUTs) are enzymes that catalyse the transfer of fucose residue from GDP‐fucose donor to acceptor substrates like proteins, glycan and polysaccharides. Previous studies showed that O‐fucosyltransferase SPY fucosylated the *Arabidopsis* DELLA protein RGA, which enhanced DELLA activity by promoting DELLA binding to a transcription factor in BR and light‐signalling pathways involved in developmental processes (Zentella *et al*., 2017). FUT1 specifically catalysed the fucosylation of xyloglucan in *Arabidopsis* (Vanzin *et al*., 2002). FUT4 and FUT6 are both arabinogalactan proteins (AGPs)‐specific FUTs with different expression patterns in both roots and leaves and differing sub‐localization in roots (Soto *et al*., 2021; Tryfona *et al*., 2014). *fut4*/*fut6* double mutants were more sensitive to salt stress with shorter roots relative to the wild type (Tryfona *et al*., 2014), which suggested that fucosylated AGPs are involved in proper cell growth under environmental stresses. FucTA and FucTB are α‐1,3‐fucosyltransferase responsible for the transfer of core α‐1,3‐linked fucose residues to glycoprotein N‐glycans. A recent study showed that several fucosyltransferases mutants, *spy*, *fut4 fut6* and *fucTa fucTb*, rather than *fut1*(*mur2*), exhibited compromised plant defence including apoplastic, stomatal defences, PTI and ETI in *Arabidopsis* (Zhang *et al*., 2019). In this study, we detected that maize *ZmFUT1* encoding protein O‐fucosyl transferase 1 was significantly associated with SLB resistance and found that *zmfut1* mutants exhibited much more susceptibility to pathogen *C. heterostrophus* (Figure 5). Those studies suggested that FUTs play important roles not only in plant development but also in plant immunity. Despite lots of researches on FUTs, the roles of FUTs in regulating plant immunity is very limited, which should be revealed in further studies.

Three genes *rhm1*, *ZmCCOMOAT2* and *ZmAPX1* resistant to SLB have been cloned (Yang *et al*., 2017; Zhang *et al*., 2022; Zhao *et al*., 2012). However, only gene *rhm1* was detected in this study, and located in two QTLs, respectively, from ZHENG58/SK (PVE, 14.7%) and KUI3/B77 RILs (PVE, 6.8%). In comparison with the results of previous studies, 67.9% (74/109) of loci identified in this study were colocalized, inferring that those populations may have different resistance genes or allele frequencies. This may also be the reason why *ZmCCOMOAT2* and *ZmAPX1* were not detected in our present study. In the present study, we identified *ZmFUT1* and *MYBR92* as the putative genes responsible for two QTLs for SLB resistance. Knockout of two genes does not significantly affect the agronomic traits. Pyramid of *ZmFUT1* and *MYBR92* genes can significantly improve SLB resistance (Figure S19), inferring that those two genes had potential applications in maize breeding.

In summary, we identified a large number of QTLs and genes responsible for SLB resistance in maize, which will facilitate functional research and genetic improvement of maize disease resistance.

## Methods

### Plant materials and field design

In this study, two genetic populations, the AMP and the ROAM, were planted at multiple locations in China. AMP population consisted of 513 maize inbred lines from temperate (TEM), tropical and subtropical origin (TST), and it was divided into TST and TEM sub‐populations (225 TST lines; 173 TEM lines: 36 SS lines, 137 NSS lines). Detailed information about those lines were described in previous studies (Li *et al*., 2013; Liu *et al*., 2017a; Yang *et al*., 2011, 2014). The AMP population was planted at Baoding (Hebei province) in 2011 and 2012 (11BD, 12BD), at Changge (Henan province) in 2011 and 2012 (11CG, 12CG) and at Xinxiang (Henan province) in 2014 (14XX). In each location, the AMP population was planted with two replicates with a randomized complete block design. For the ROAM population, it consisted of eight RIL families (B73/BY804, BY815/KUI3, K22/BY815, K22/CI7, KUI3/B77, YU87‐1/BK, ZHENG58/SK, ZONG3/YU87‐1), which derived from 12 parents (B73, B77, BK, BY804, BY815, CI7, K22, KUI3, SK, YU87‐1, ZHENG58, ZONG3). A total of 12 parental lines were also part of the AMP population. Each of those eight RIL families was derived from a single F_1_ plant and was constructed by continuously self‐crossing and single seed descent method for at least six generations. Eight RIL families comprised of 1540 lines and an average of 192 lines per RIL, which ranged from 165 to 207 (Table S1). Two RIL families (K22/BY815, ZONG3/YU87‐1) were planted with two replicates at two locations Changge and Baoding in 2012. All eight RIL families of ROAM were planted with one replicate along with the parental lines of each RIL grown before and after the corresponding RIL family at Xunxian (Henan province) in 2012. For ROAM and AMP populations, 11 plants of each line were grown in a single 3‐m row spaced 0.67 m apart. Sprinkling irrigation and standard agricultural practices were used to ensure satisfactory plant growth.

### Phenotypic investigation and statistical analysis

*Cochliobolus heterostrophus* was isolated from susceptible lines collected in previous seasons. The inoculum of *C. heterostrophus* was cultured on potato dextrose agar (PDA) medium. To produce enough inoculum, inocula were transferred to sterile sorghum (*Sorghum biocolor*) kernels following culturing at room temperature (25 °C) for 2–3 weeks. Conidia were washed from sorghum kernels with sterile water and 1 drop of Tween 20 per 100 mL was added to the suspension solution. Plants were inoculated at the V7‐V8 growth stage by spraying suspension. Two weeks after flowering, the SLB severity was scored. According to the percentage of necrotic leaf area on a whole plant leaf, disease scores were rated from 1 to 9 scale that ‘1’ as the most resistant phenotype and ‘9’ as the most susceptible phenotype. Rating ‘1’ indicates no disease symptoms on a leaf. Rating ‘2’, ‘3’, ‘4’, ‘5’, ‘6’, ‘7’, ‘8,’ respectively, means 0%–5%, 5%–25%, 25%–40%, 40%–50%, 50%–60%, 60%–75%, 75%–90% of leaf area infected. Rating ‘9’ means the whole plant was infected and dying.

For the ROAM population at Xunxian in 2012, 2 weeks after flowering, five RIL families, except three RILs (YU87‐1/BK, K22/CI7 and ZHENG58/SK), were scored for SLB severity. Two weeks later, the entire ROAM population were again investigated for SLB score. At those two time‐points, the parental lines of each RIL were also investigated, and the average value of parental lines between two time‐points investigations was used for subsequent analysis. For two RIL families (K22/BY815 and ZONG3/YU87‐1) at Changge and Baoding locations in 2012, all lines were investigated at 4 weeks after flowering. The best linear unbiased prediction (BLUP) value of each line was calculated across all environments and two time‐points. BLUP values were used to give an SLB index for the final QTL mapping. For K22/BY815 and ZONG3/YU87‐1 RIL families, three environmental phenotypes (two replicates at Changge, two replicates at Baoding and two time‐points at Xunxian) were used for calculating BLUP value and broad‐sense heritability. For three RIL families (B73/BY804, KUI3/B77 and BY815/KUI3), the two time‐point phenotypes were used for calculating BLUP value and repeatability heritability. For three RIL families (YU87‐1/BK, K22/CI7 and ZHENG58/SK), the one time‐point phenotype was used as the SLB index for subsequent analysis. For the AMP population, the SLB score for each inbred line was collected at 4 weeks after flowering and calculated as the average of its original value for two replicates in each location. The average SLB score in a single location and BLUP value across all environments were both used as the SLB index for final statistical analysis.

The heritability of the SLB index was calculated using the formula: $H^{2}=\delta_{g}^{2}/\left(\delta_{g}^{2}+\delta_{e}^{2}/n\right)\delta_{g}^{2}/\left(\delta_{g}^{2}+\delta_{e}^{2}/n\right)$, where $\delta_{g}^{2}$ is the genotypic variance, $\delta_{e}^{2}$ is the residual error variance and *n* is the number of environments. Pearson correlation was used in R(version 3.5.1, R Foundation for Statistical Computing: Vienna, Austria) to determine the correlations of the AMP SLB index across all different environments.

### QTL mapping in ROAM population

The previously reported eight RIL families of ROAM (Chen *et al*., 2016; Liu *et al*., 2017b; Wang *et al*., 2018; Xiao *et al*., 2016) were genotyped using Illumina MaizeSNP50 BeadChip. A total of 11 360–15 285 high‐quality polymorphic markers for each RIL family were used to construct high‐density genetic maps (Pan *et al*., 2016). Those RIL families were covered with an average of 2436 unique bins that contain no recombination events. The agronomic traits of ROAM population contained plant architecture and yield‐related traits (Table S13), and the detailed information was referred to previous studies (Liu *et al*., 2017b; Pan *et al*., 2017; Xiao *et al*., 2016).

For the SLM method, composite interval mapping was implemented in Windows QTL Cartographer 2.5 for QTL mapping (Zeng, 1994). The program was run with default parameters and walk speed = 1.0 cM. The cut‐off was set as LOD = 3.0, and the two‐LOD drop interval from the QTL peak was defined as the QTL support interval. If the QTL regions of different populations overlapped with more than a 1 Mb interval, those QTLs were defined as consensus QTLs, and the remaining non‐overlapping QTLs were regarded as unique QTLs in each population. The QTL that explained phenotypic variation of more than 10% was defined as a major QTL. The JLM and GWAS methods in the ROAM population were implemented as described (Xiao *et al*., 2016). For JLM, a linear mixed model and the restricted maximum likelihood were used to detect the significant recombination blocks, where marker and polygenic effects were set as random effects, the population mean and intercept term were treated as fixed effects. A permutation test of 500 permutated samples was conducted to calculate the threshold of LRT scores. The threshold of LRT was 2.76 at the Type I error rate of 0.05, and the physical interval with LRT ≥ 2.76 was the JLM QTL support region. For GWAS, stepwise regression was performed on whole RIL datasets to identify significant SNPs. SNPs with resample model inclusion probability ≥0.02 were regarded as significant SNPs. To reduce the SNP redundancy, a final backward regression was employed for testing significant SNPs identified by stepwise regression and resample analysis. The mean threshold *P*‐value of ten chromosomes was used as the cutoff of the final backward model. Significant SNPs included in the final backward regression model were considered as candidate SNPs for the SLB index.

For SLM QTLs and significant loci of GWAS from ROAM and AMP populations, the corresponding bins were selected from the integrated genetic maps of ROAM population based on the peak position of QTLs/loci. When the overlapped loci identified with multiple methods had different peak bins, one peak bin was selected, prior to results from JLM or GWAS. The additive allelic effects of founders underlying those peak bins were estimated by fitting final trait models using the ‘lm’ function within the ‘lme4’ package in R, which also calculates the significance of each effect in two‐sided independent *t*‐tests. A threshold of false discovery rate at 0.05 was used to define significant allele effects across founders within each QTL. The predictive value of the QTL model was evaluated by predicting the SLB index values of 12 founder lines. Founder values were predicted by summing their respective RIL family average value and their corresponding allelic effect at all 109 allelic effects, multiplying by a factor of two and adding this sum to the intercept of the joint linkage model. The prediction power between the observed SLB index and the model prediction values of founder lines was evaluated with linear regression.

### GWAS in AMP population

The 513 inbred lines of the AMP population were genotyped by deep RNA‐seq, GBS, and various arrays with densities of 50 K (MaizeSNP50 BeadChip) and 600 K (Affymetrix Axiom Maize Genotyping 600 K Array; Fu *et al*., 2013; Liu *et al*., 2017a). In total, 2.65 M SNPs were obtained from the AMP population and 1.25 M SNPs (MAF ≥ 0.05) were used for the following analyses. Detailed information about the genotype dataset was described in previous studies (Liu *et al*., 2017a). The phenotypic data of AMP containing flowering time, plant architecture and yield‐related traits were referred to in a previous study (Yang *et al*., 2014). The genetic relationships between ROAM and AMP populations were visualized by the principal component analysis (PCA). Two populations were both genotyped by using MaizeSNP50 BeadChip, and therefore a total of 3234 SNPs with MAF ≥ 0.4 were selected and used to conduct PCA.

Genome‐wide association study was performed by using a mixed linear model implemented in TASSEL software, where population structure (Q) and kinship matrix (K) were taken into account for controlling false‐positive associations (Bradbury *et al*., 2007; Yu *et al*., 2006). To avoid false negatives, the adjusted Bonferroni‐corrected threshold was used to determine the GWAS threshold, as *P* = 1/*n*_e_, where *n*_e_ is the number of independent makers (Wang *et al*., 2016). The number of independent markers was determined by PLINK with *r* ^*2*^ = 0.2 and 50‐window size, resulting in the GWAS threshold of 5.63 × 10^−6^ (1/177 656). For simplicity, the threshold was set as 1 × 10^−5^. The SNP with the lowest *P* value in each signal was selected as the peak SNP. If the plant defence‐related gene is located within 100 Kb up‐ and downstream region of peak SNP and expressed in the leaf tissue, the defence gene was considered as the most likely candidate gene. If there were no annotated defence‐related genes, the gene that was induced by the pathogen would be considered as the most likely candidate gene. When none of these criteria was met, the closest gene under peak SNP was considered as the most likely candidate gene. The corresponding gene and physical position of SNPs were identified from maize inbred line B73 reference genome version (AGPv2, FGS 5b; Schnable *et al*., 2009). The function annotation and gene expression pattern of candidate genes in the B73 inbred line were retrieved from the MaizeGDB website (https://www.maizegdb.org/gene_center/gene). Additional annotations about GO description and GO term were obtained from agriGO v2.0 (Tian *et al*., 2017) and Gene Ontology terms (http://geneontology.org/), which were used to manually classify genes into functional categories.

We analysed ROAM QTLs in the AMP population: firstly 66 ROAM QTLs were mapped into the AMP population based on the interval of peak bins. Then the most significant SNPs were selected from those intervals and regarded as tagged SNPs for those ROAM QTLs. Subsequently, the resistant/susceptible alleles were determined with the SLB index for 109 QTLs in the 100 kb flanking region of tagged SNPs. At last, the allele of each line at each locus was determined by resistant allele frequency between each line and all inbred lines. When two criteria that peak SNP carry the resistant allele and the resistant allele frequency is higher than the mean value of all inbred lines in the 100 kb flanking region of peak SNP were met, lines were considered to carry the resistant allele at this locus. The association networks were constructed using the program gephi (version 0.9.2, Bastian *et al*., 2009) with SLB resistance, agronomic traits and resistant loci as nodes, and association significance between traits and loci as edges. The fixation index (*F*_ST_) was calculated among tropical and temperate lines using Vcftools considering all SNP markers with MAF ≥ 0.1. Only those SNPs located in the 100 Kb flanking region of 109 tagged SNPs were shown.

### Overlapped QTL

When JLM QTL covered the SLM QTL region with more than 1 Mb or located in its region, those QTLs were considered as overlapped QTLs. When the peak SNPs of ROAM GWAS loci were located in SLM or JLM QTL interval, the numbers of peak SNPs were counted and the GWAS loci were co‐located with SLM or JLM QTL region.

To ascertain the overlap between significant signals of different environments identified via GWAS in the AMP population, the distance between peak SNPs in different environments was <700‐Kb to be considered as consensus loci. In order to compare with the ROAM QTLs identified in the present study, the peak SNPs were checked one by one whether those SNPs located in the QTL interval identified via SLM and JLM and the 1‐Mb flanked region of peak bin identified via final backward regression model. The comparison of entire 109 QTLs with previous studies was conducted as followed: first, we collected QTLs information from ten studies of individual biparental populations or loci/SNPs in association population (Balint‐Kurti *et al*., 2006, 2007; Balint‐Kurti and Carson, 2006; Carson *et al*., 2004; Kump *et al*., 2011; Lennon *et al*., 2017; Liu *et al*., 2011; Lopez‐Zuniga *et al*., 2019; Negeri *et al*., 2011; Zwonitzer *et al*., 2009). Second, we placed QTLs or loci on the B73 reference genome (AGPv2 5b version) according to the positions of their closest flanking markers or support intervals. Third, the comparison of 109 QTLs interval of the present study with those reported QTLs interval or the 1‐Mb flanking region of 51 significant SNPs in the NAM population was used to determine whether those QTL intervals were co‐located.

### Analysis of epistatic QTL for SLB

The peak SNPs identified in the ROAM and AMP populations were used for epistatic interaction analysis. A linear model regression analysis was used as follows: *y* = *u* + *g*_i_ + *g*_j_ + *g*_i_*g_j_, where *y* is the phenotype of the SLB index, *g*_i_ and *g*_j_ are the main effects associated with loci i and j respectively, *g*_i_*g_j_ are the interaction effects between alleles at the loci i and j. All pair‐wise interactions were tested and significant epistatic interactions were filtered by the threshold *P* value <0.05/*N* (*N*, total numbers of all pairwise epistatic interactions among significant SNPs). The epistatic interactions were only detected in the 11CG environment in the AMP population. The proportion of phenotypic variance due to epistatic interactions was determined by comparing the residual of the above full model with that of the additive model *y* = *u* + *g*_i_ + *g*_j_. This analysis was implemented with scripts in R version 3.5.1.

### RNA extraction and real‐time quantitative PCR

Total RNA was isolated from maize leaf samples by TRIzol Reagent (Invitrogen) following the manufacturer's instructions. cDNA was synthesized from the 2 μg RNA using the TransScript One‐Step gDNA Removal and cDNA synthesis SuperMix (TransGen Biotech, China). Quantitative real‐time PCR (qRT‐PCR) was performed on the Bio‐Rad CFX96 Touch Real‐Time PCR detection system using AceQ qPCR SYBR Green Master Mix (Vazyme). Primers ChActin (Forward: TCAAGATCATCGCTCCTCCC; Reverse: GGACCGCTCTCGTCGTACTC) was used to amplify *ChACT1* gene of *C. heterostrophus*. Primers ZmActin (Forward: TACGAGATGCCTGATGGTCAGGTCA; Reverse: TGGAGTTGTACGTGGCCTCATGGAC) was used to amplify maize *ZmACTIN* internal control. To detect the gene expression level in the B73 inbred line after inoculation with *C. heterostrophus*, primers qFUT1 (Forward: AGAAGCTTGGTGTTACTGACG; Reverse: CTTAACCTCTGCAGGCTTCCA) were used to amplify *ZmFUT1* gene. To quantify fungal biomass, the relative transcript level between *ChACT1* and *ZmACTIN* was calculated with the $2^{-\Delta \Delta C_{t}}$ method. For each sample, qPCR was carried out in three biological replicates, with three technical replicates for each biological replicate.

### Identification of candidate genes of two QTLs for SLB resistance

For JLM QTL 1_279.42 containing the *ZmFUT1* gene, this QTL was co‐located with SLM QTLs in K22/BY815 and ZONG3/YU87‐1 RIL families. At this region, another small effect QTL (LOD 2.34; PVE 4.99%) that was detected in BY815/KUI3 RIL family was taken into consideration, although it was below the significant threshold. To identify candidate genes of two JLM QTLs (1_279.42 and 4_34.73), we integrated with multiple public data in the present study. We used high‐density markers, SNPs and InDels, from a previous study (Yang *et al*., 2019), to conduct candidate gene association analysis. To infer the functional mechanisms of candidate genes, we used the gene expression data in the AMP population for leaf tissue by RNA sequencing from the previous studies (Liu *et al*., 2020b). To determine gene expression after *C. heterostrophus* pathogen inoculation, we collected two samples of B73 seedlings at each time point, 0 and 36 h after inoculation, to do RNA sequencing. The RNA‐seq reads were mapped to the B73 v4 genome using TopHat2 with default parameters. The gene expression abundances were determined with Cufflinks with default parameters. For haplotype‐based association analysis, we tested the lines, carrying all resistant alleles, versus the remaining lines for each combination by using Student's *t*‐test in the AMP population.

### CRISPR/Cas9 editing experiment

To validate *ZmFUT1* and *MYBR92* as the causal gene responsible for QTL 1_279.42 and 4_34.73, respectively, we designed two guide RNA (gRNAs) sequences to edit the first gene exon against each gene. Two gRNAs were cloned into a CRISPR/Cas9 plant expression vector (Liu *et al*., 2020a) and transformed into inbred line KN5585 by Agrobacterium‐mediated transformation at the WIMI Biotechnology Co., Ltd. The Primers (forward: CCGTGCCCGATAAATAAGAA, reverse: GAAATGGCACCTTTCGACAT) were designed to amplify about 548 bp for genotyping of *mybr92* mutants. Primers (forward: GGTTGACTTGCTTCAGCCTATCAA, reverse: TATGGCTGCCGATCGTAGACG) were used to amplify 714 bp for genotyping of *zmfut1* mutants. The PCR products were used for Sanger sequencing to examine the sequence variations. The maize knockout lines and wild‐type lines were planted in a greenhouse, and at 3‐week‐old seedling, those lines were sprayed inoculation with 50 mL of *C. heterostrophus* conidial suspensions (2.5 × 10^4^ per mL) and sealed with plastic bags to maintain moisture for 14 h. Necrotic lesion formation was observed at 3 days later on the leaf. The SLB severity was investigated based on the percentage of necrotic leaf area on the third leaf by using 1–9 ratings. Those maize knockouts and wild‐type lines were grown in three environments, one location at Huanggang (22HG) and two locations at Wuhan (22WH1, 22WH2), Hubei province, China in 2022. The agronomic traits (flowering time, plant architecture and yield‐related traits) were investigated. A statistically significant difference between wild‐type lines and mutants was evaluated by a two‐tailed Student's *t*‐test with a *P* value <0.05.

## Conflict of interest

The authors declare that they have no competing financial interests.

## Author contributions

J.Y. and J.D. designed and supervised this research. J.Y. and J.D. designed the experiments for RIL families. J.Y. and B.L. designed the experiments for the AMP population. S.D., W.L., X.W. and J.D. managed the field experiments. X.W. and J.D. collected the field phenotyping data. G.C. and Z.D. collected the transgenic phenotyping data. G.C., Y.X., J.D., Z.L., and B.L. performed the data analysis. G.C. and J.Y. prepared the manuscript. J.D., B.L. and J.S.J. edited the manuscript.

## Supporting information


**Figure S1** The phenotypic distribution of resistance to southern corn leaf blight across the ROAM RIL families.
**Figure S2** The phenotypic distribution of SLB resistance in the AMP population.
**Figure S3** Phenotypic variation of SLB resistance in sub‐populations of AMP population.
**Figure S4** Percentage of QTL number and interval identified by SLM and JLM.
**Figure S5** The overlapped QTLs identified by three methods in ROAM population.
**Figure S6** Manhattan plot and quantile‐quantile plot for SLB index of five environments and BLUP.
**Figure S7** Comparison of the reported QTLs identified in previous studies with QTLs detected in ROMA and AMP populations of this study.
**Figure S8** The allele effects of 109 QTLs for 12 founder lines.
**Figure S9** The distribution of resistant alleles in the AMP population.
**Figure S10** The resistant allele frequency and selection signatures of 109 QTLs.
**Figure S11** Effects of 37 resistant alleles on agronomic traits.
**Figure S12** The influence of 47 resistant and non‐adapted loci on agronomic traits.
**Figure S13** The resistant allele of chr6.S_151638555 affected multiple agronomic traits.
**Figure S14** Expression analysis of *ZmFUT1*.
**Figure S15** The fungal biomass of wild‐type and *zmfut1*‐knockout lines inoculated with *C. heterostrophus* pathogen.
**Figure S16** The agronomic traits of *zmfut1*‐knockout lines.
**Figure S17** The fungal biomass of wild‐type and *mybr92*‐knockout lines inoculated with *C. heterostrophus* pathogen.
**Figure S18** The agronomic traits of *mybr92*‐knockout lines.
**Figure S19** Haplotype‐based association between *ZmFUT1* and *MYBR92*.

**Table S1** Mean, standard deviations, variation ranges and broad‐sense heritability for SLB index in the ROAM RIL families.
**Table S2** Mean, standard deviations, variation ranges, correlation and broad‐sense heritability for SLB index in the AMP population.
**Table S3** The QTL information for SLB resistance through the SLM method in the ROAM population.
**Table S4** Summary of SLM QTLs for SLB resistance in the ROAM population.
**Table S5** The QTL information for SLB resistance through the JLM method in the ROAM population.
**Table S6** All significant SNPs for SLB resistance through GWAS in the ROAM population.
**Table S7** A total of 19 significant SNPs for SLB resistance included in the final GWAS model in the ROAM population.
**Table S8** Significant pair‐wise epistatic interactions between loci and their explained phenotypic variances.
**Table S9** The significant SNPs for SLB resistance in five environments and BLUP in the AMP population.
**Table S10** A total of 61 SNPs detected as significantly associated with SLB resistance and the function and GO information of candidate genes.
**Table S11** Significant pair‐wise epistatic interactions between loci and their explained phenotypic variances in the AMP population.
**Table S12** A total of 109 QTLs identified in ROAM and AMP populations and corresponding information for candidate genes.
**Table S13** The correlation between SLB resistance and agronomic traits in ROAM and AMP populations.
**Table S14** The frequency of resistant alleles underlying 109 QTLs in the tropical and temperate sub‐populations.
