# Supplementary material for: Genetic basis of resistance to southern corn leaf blight in the maize multi‐parent population and diversity panel
Source: Plant Biotechnol J. 2023 Jan 9;21(3):506–20. doi: 10.1111/pbi.13967 (PMC9946143; doi:10.1111/pbi.13967)
Supplement: Supplementary file 1 — Figure S1 The phenotypic distribution of resistance to southern corn leaf blight across the ROAM RIL families. Figure S2 The phenotypic distribution of SLB resistance in the AMP population. Figure S3 Phenotypic variation of SLB resistance in sub‐populations of AMP population. Figure S4 Percentage of QTL number and interval identified by SLM and JLM. Figure S5 The overlapped QTLs identified by three methods in ROAM population. Figure S6 Manhattan plot and quantile‐quantile plot for SLB index of five environments and BLUP. Figure S7 Comparison of the reported QTLs identified in previous studies with QTLs detected in ROMA and AMP populations of this study. Figure S8 The allele effects of 109 QTLs for 12 founder lines. Figure S9 The distribution of resistant alleles in the AMP population. Figure S10 The resistant allele frequency and selection signatures of 109 QTLs. Figure S11 Effects of 37 resistant alleles on agronomic traits. Figure S12 The influence of 47 resistant and non‐adapted loci on agronomic traits. Figure S13 The resistant allele of chr6.S_151638555 affected multiple agronomic traits. Figure S14 Expression analysis of ZmFUT1. Figure S15 The fungal biomass of wild‐type and zmfut1‐knockout lines inoculated with C. heterostrophus pathogen. Figure S16 The agronomic traits of zmfut1‐knockout lines. Figure S17 The fungal biomass of wild‐type and mybr92‐knockout lines inoculated with C. heterostrophus pathogen. Figure S18 The agronomic traits of mybr92‐knockout lines. Figure S19 Haplotype‐based association between ZmFUT1 and MYBR92. [file PBI-21-506-s002.pdf]

Supporting figures

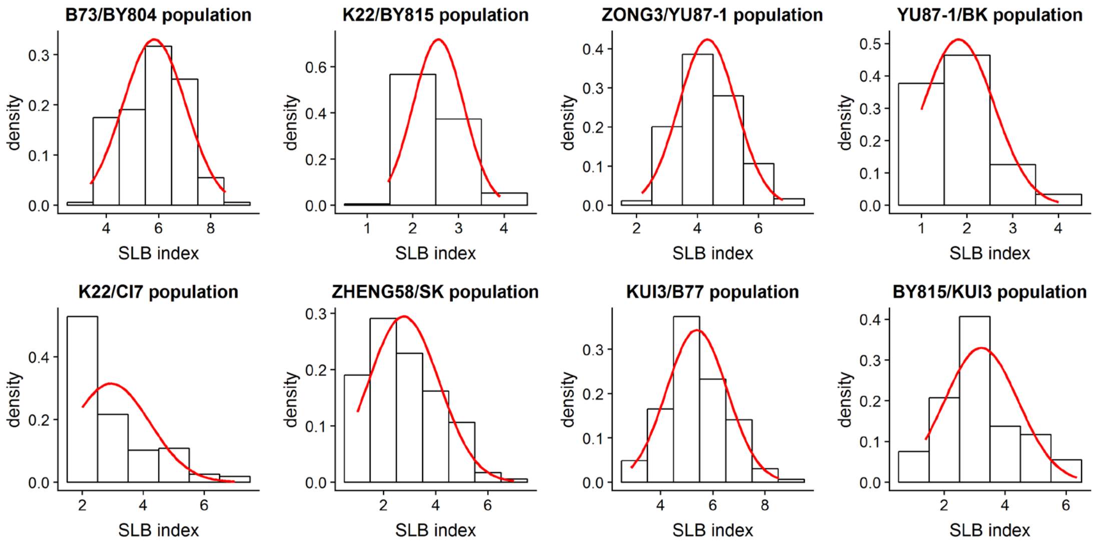

Figure S1 The phenotypic distribution of resistance to southern corn leaf blight across the ROAM RIL families.

SLB index presented as best linear unbiased predictor (BLUP) scores, except for three RIL families (YU87-1/BK, K22/CI7, ZHENG58/SK).

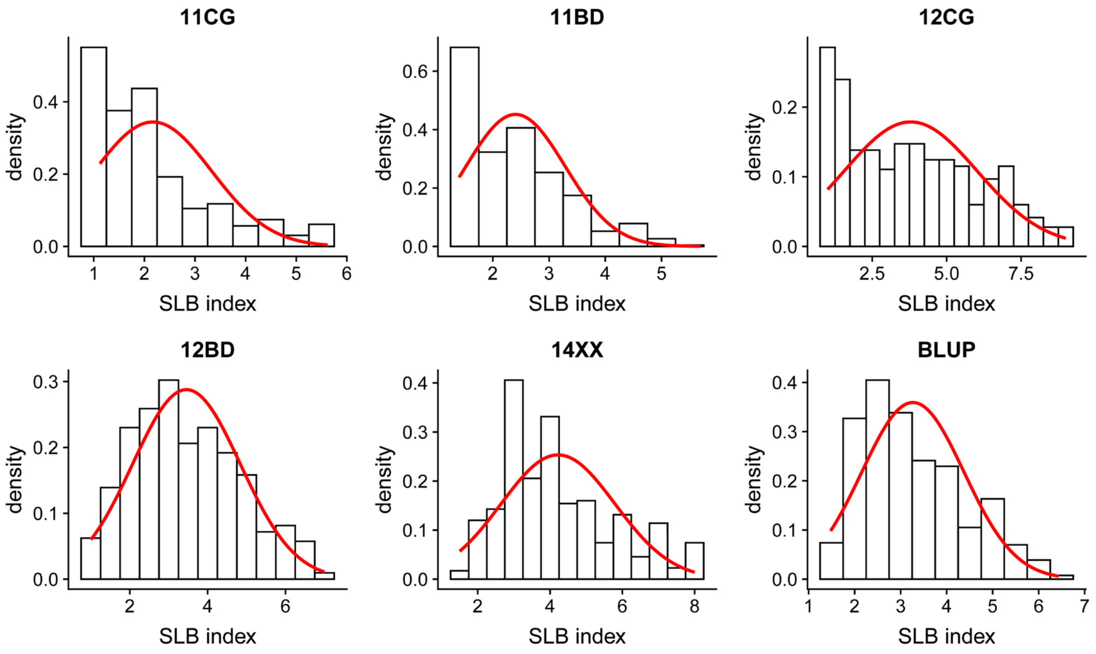

Figure S2 The phenotypic distribution of SLB resistance in the AMP population.

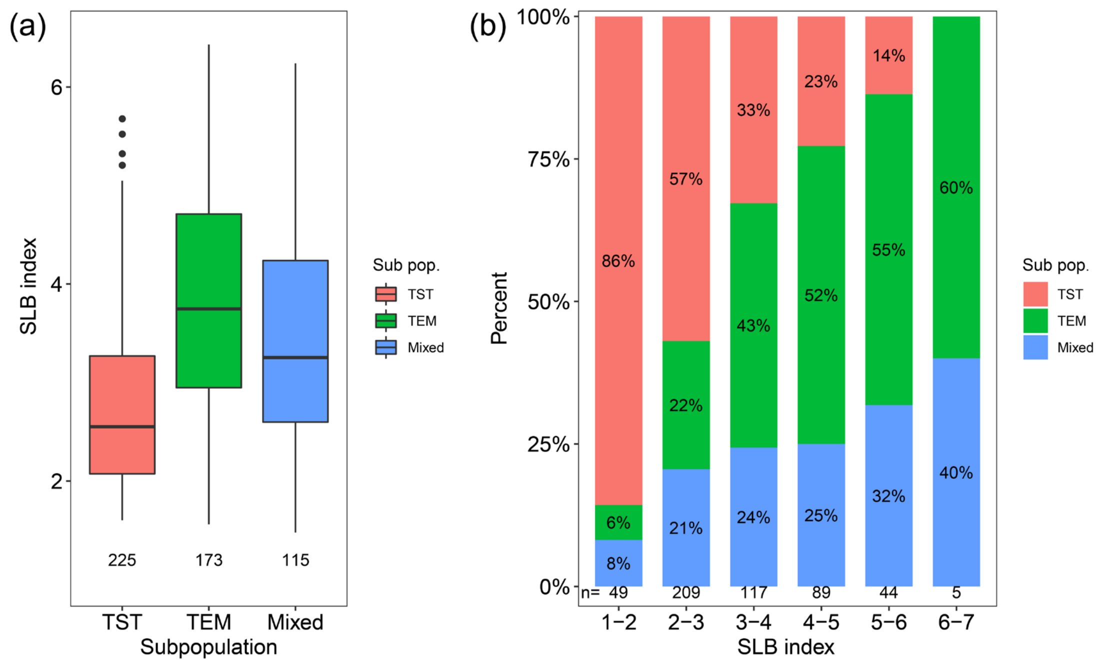

Figure S3 Phenotypic variation of SLB resistance in subpopulations of AMP population.

(a) Boxplot of SLB resistance in AMP subpopulations. Total numbers of each group were below the box. TST, tropical lines. TEM, temperate lines. (b) The stocked barplot of SLB resistance in subpopulations. Total numbers of each group were below the bar.

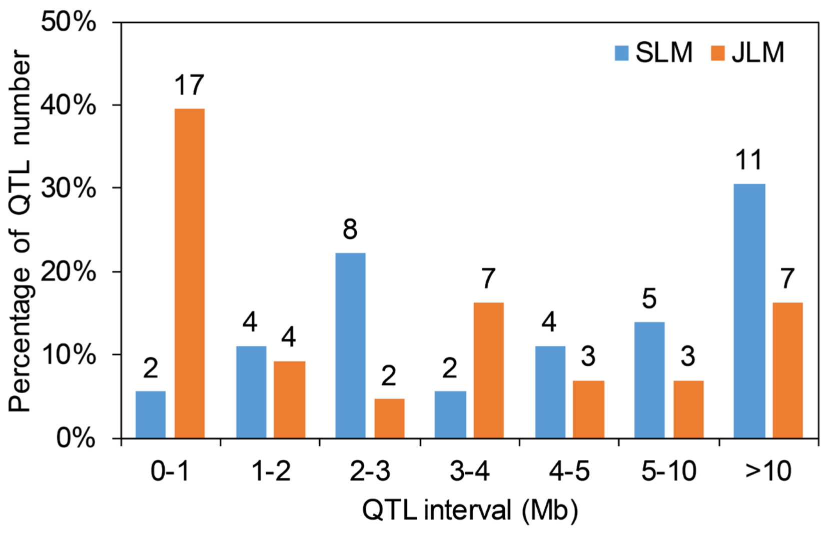

Figure S4 Percentage of QTL number and interval identified by SLM and JLM.

The QTL number of each group above the bar. Blue color: SLM method. Yellow brown: JLM method.

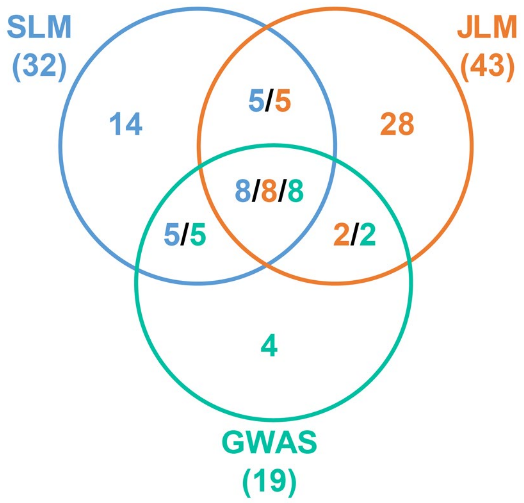

Figure S5. The overlapped QTLs identified by three methods in ROAM population.

The overlapped QTLs were identified by three methods, SLM, JLM and GWAS in ROAM population. The colored numbers are corresponding to method, blue for SLM, yellow brown for JLM, cyan for GWAS.

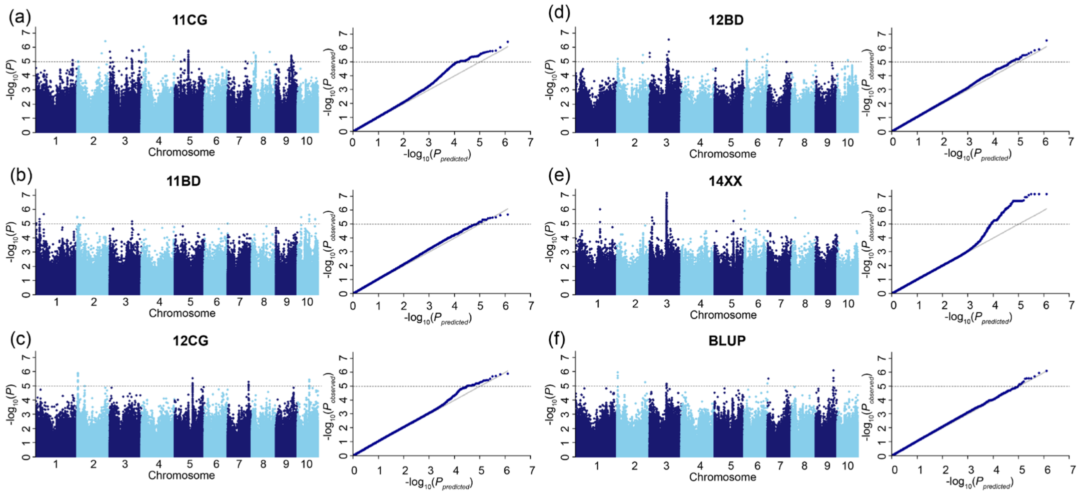

Figure S6 Manhattan plot and quantile-quantile plot for SLB index of five environments and BLUP.

(a) 11CG; (b) 11BD; (c) 12CG; (d) 12BD; (e) 14XX; (f) BLUP.

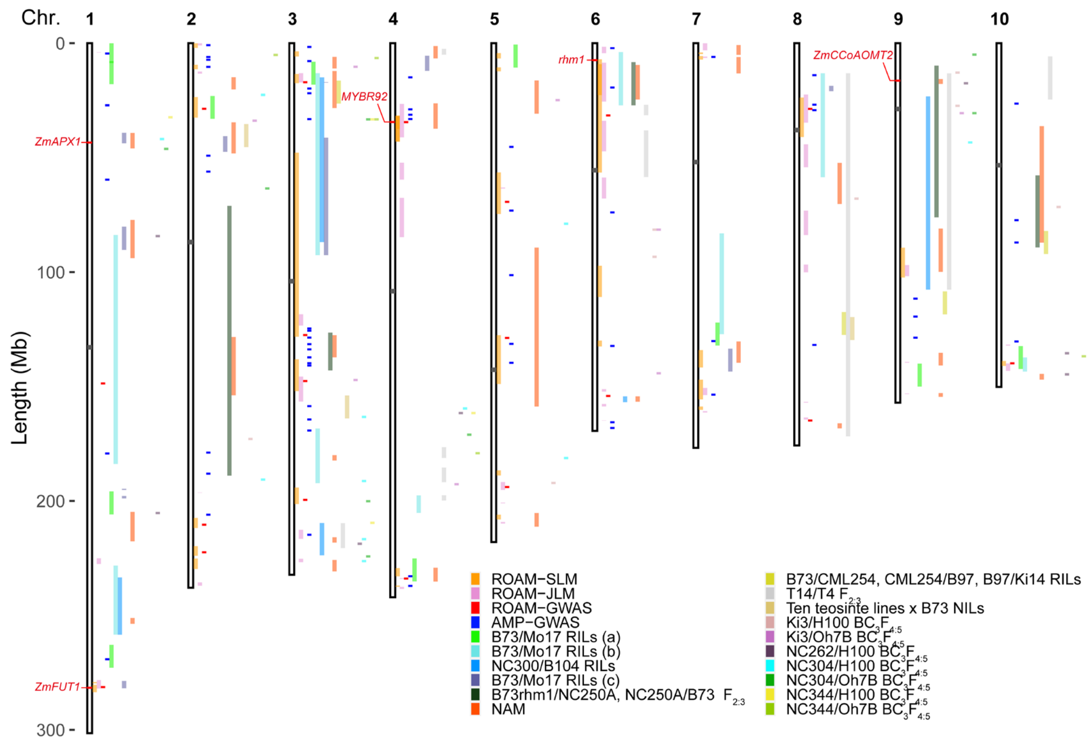

Figure S7 Comparison of the reported QTLs identified in previous studies with QTLs detected in ROMA and AMP populations of this study.

The QTLs identified in ROAM and AMP populations in this study and the reported QTLs are represented as rectangles or lines with different colors next to each chromosome. The red horizontal lines on the chromosome 1, 4, 6 and 9 represented genes *ZmAPX1*, *ZmFUT1*, *MYBR92*, *rhm1* and *ZmCCoAOMT2* respectively. Those genetic populations were referred as followed: B73/Mo17 RILs (a) (Carson et al., 2004); B73/Mo17 RILs (b) (Balint-Kurti and Carson, 2006); NC300/B104 RILs (Balint-Kurti et al., 2006); B73/Mo17 RILs (c) (Balint-Kurti et al., 2007); B73rhm1/NC250A, NC250A/B73 $F_{2:3}$ (Zwonitzer et al., 2009); NAM (Kump et al., 2011); B73/CML254, CML254/B97, B97/Ki14 RILs (Negeri et al., 2011); T14/T4 $F_{2:3}$ (Liu et al., 2011); Ten teosinte lines × B73 NILs (Lennon, 2017); Ki3/H100, Ki3/Oh7B, NC262/H100, NC304/H100, NC304/Oh7B, NC344/H100 , NC344/Oh7B $BC_3F_{4:5}$ (Lopez-Zuniga et al., 2019).

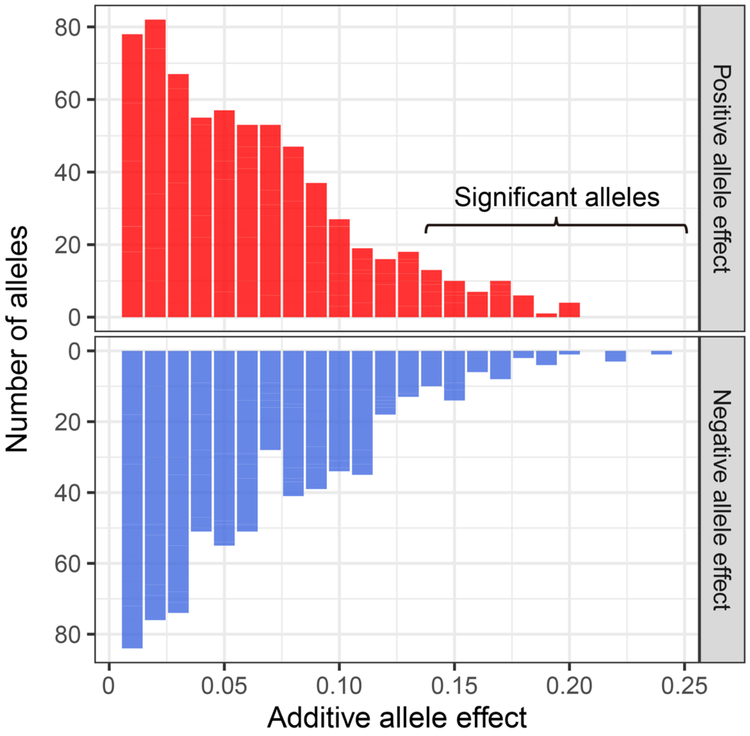

Figure S8 The allele effects of 109 QTLs for 12 founder lines.
Histogram plot of QTL additive allele estimates for all 12 founder lines relative to mean value. Count of effects decreasing SLB resistance above the line, and count of effects increasing SLB resistance below the line. Alleles labeled as significant alleles were significant at the 5% false discovery rate.

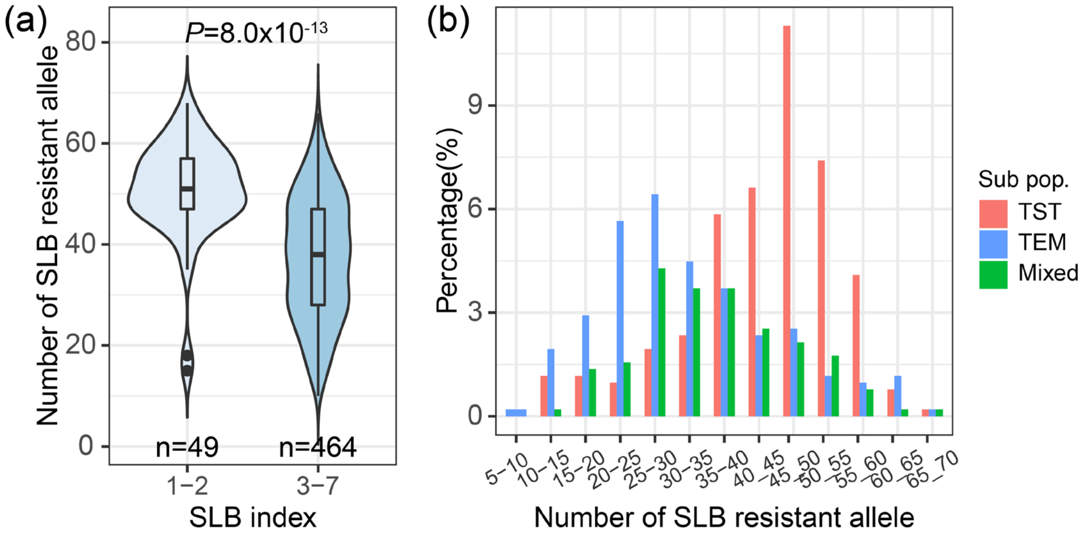

Figure S9 The distribution of resistant alleles in AMP population.
(a) The number of resistant alleles in the high resistant varieties. (b) Proportion of number of SLB resistant alleles in subpopulation.

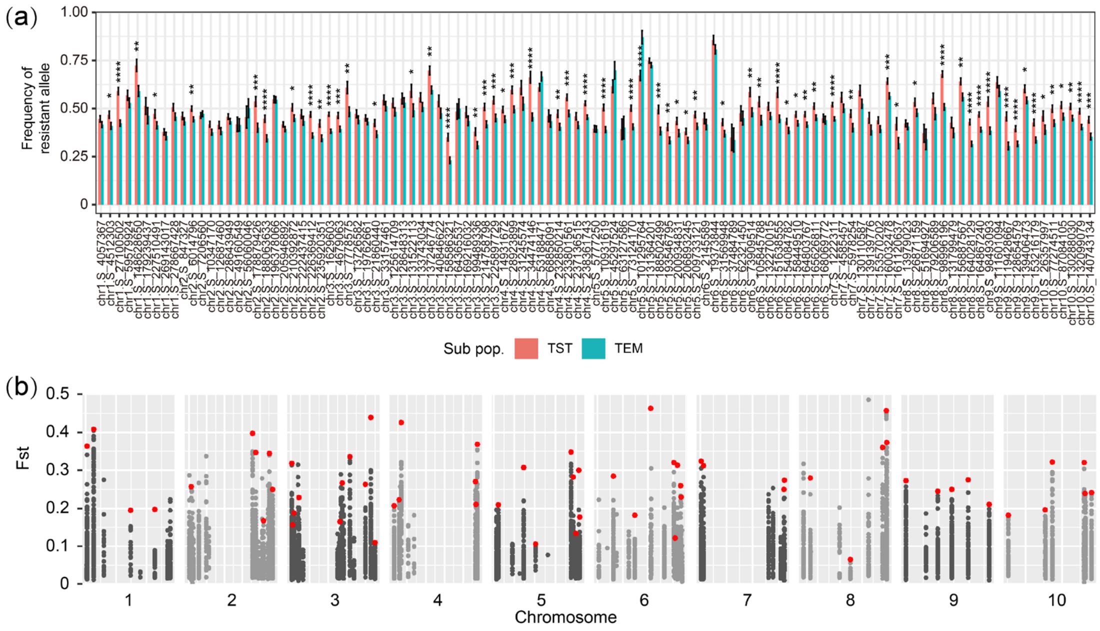

Figure S10 The resistant allele frequency and selection signatures of 109 QTLs.

(a) The plot displayed the resistant allele frequency of 109 QTLs between tropical (TST) and temperate (TEM) subpopulations. The differences were analyzed by Student's two-sided t-test, * *P*<0.05; ** *P*<0.01; *** *P*<0.001; **** *P*<0.001. (b) Manhattan plot for population fixation index (F_{ST}) of 109 loci between TST and TEM subpopulations. Those SNPs with the lowest F_{ST} value at the 62 enrichment loci were marked with red points.

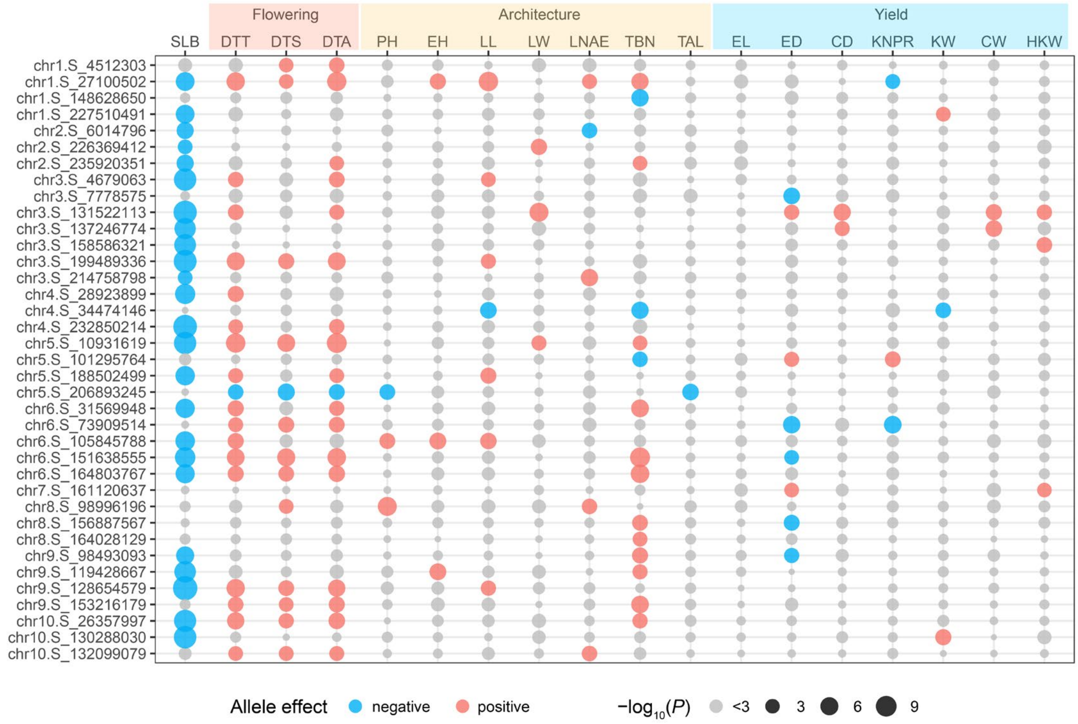

Figure S11 Effects of 37 resistant alleles on agronomic traits.

Allele effects of resistant allele relative to susceptible allele were shown with sky blue (negative) and light red colors (positive) for SLB resistance and agronomic traits. The dot size represented the degree of significance (-log$_{10}P$ value). DTT, days to tasseling; DTA, days to anthesis; DTS, days to silking; PH, plant height; EH, ear height; LL, ear leaf length; LW, ear leaf width; LNAE, leaf number above ear; TBN, tassel branch number; TAL, tassel main axis length; EL, ear length; ED, ear diameter; CD, cob diameter; KNPR, kernel number per row; KW, kernel width; CW, cob weight; HKW, hundred kernel weight.

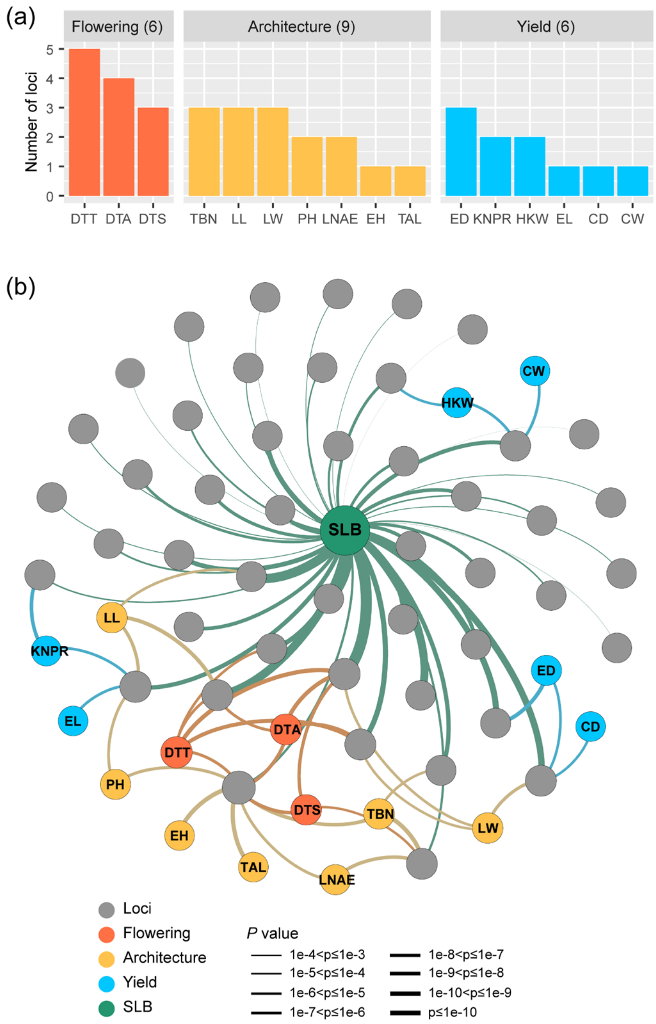

Figure S12 The influence of 47 resistant and non-adapted loci on agronomic traits.
(a) Number of resistant loci linked to agronomic traits. The total number of loci affecting flowering time, plant architecture and yield traits were shown in brackets. (b) Association network among SLB resistance and agronomic traits in AMP. The nodes represent SLB resistance and agronomic traits, and the SLB resistant loci. The edges between loci and different traits are linked by the significance. 47 non-adapted loci were displayed.

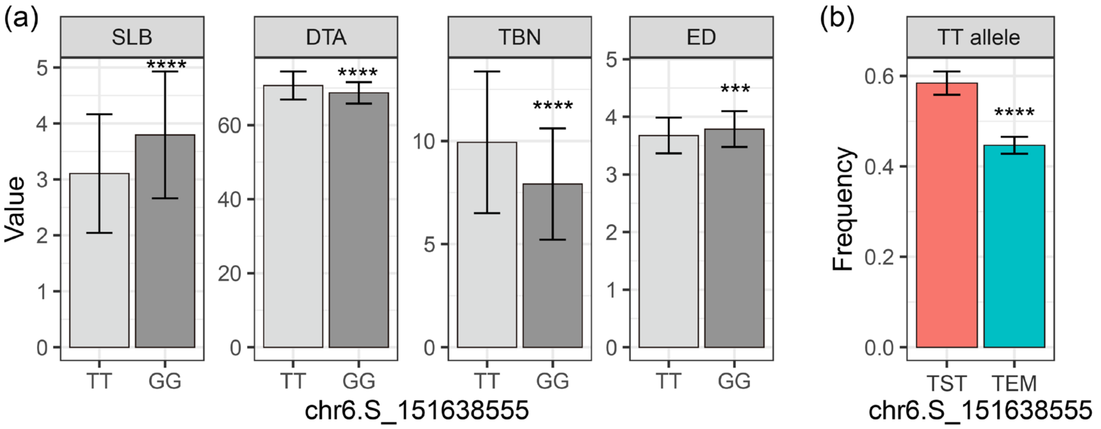

Figure S13 The resistant allele of chr6.S_151638555 affected multiple agronomic traits.

(a) The allele effect of chr6.S_151638555 on SLB resistance and agronomic traits. The differences were analyzed by Student's two-sided *t*-test, *** *P*<0.001; **** *P*<0.001. (b) Difference of allele frequency between TST and TEM lines at chr6.S_151638555.

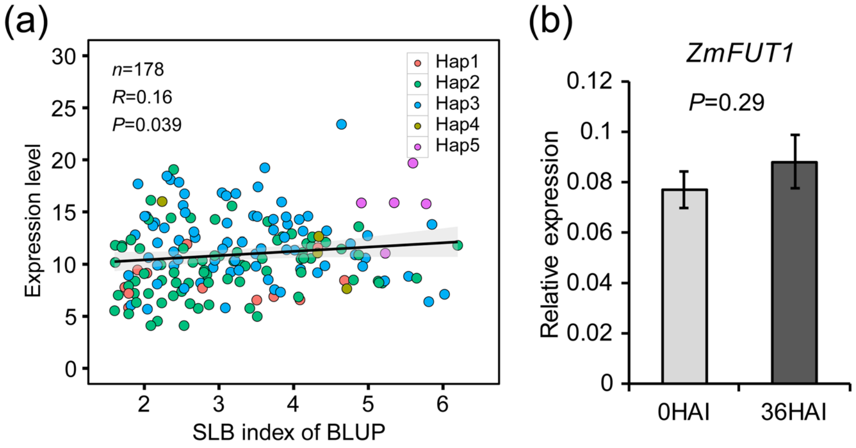

Figure S14 Expression analysis of *ZmFUT1*.

(a) Correlation between gene expression of *ZmFUT1* and SLB index in the AMP population. The regression curves and correlation coefficient were displayed. (b) Gene expression of *ZmFUT1* was assessed by qPCR in inbred line B73 inoculated with *C. heterostrophus*.

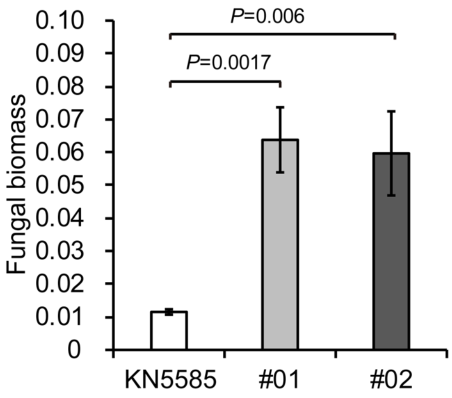

Figure S15 The fungal biomass of wildtype and *zmfut1*-knockout lines inoculated with *C. heterostrophus* pathogen.

Values are means ± SD. Differences among groups were analyzed by Student's *t*-test.

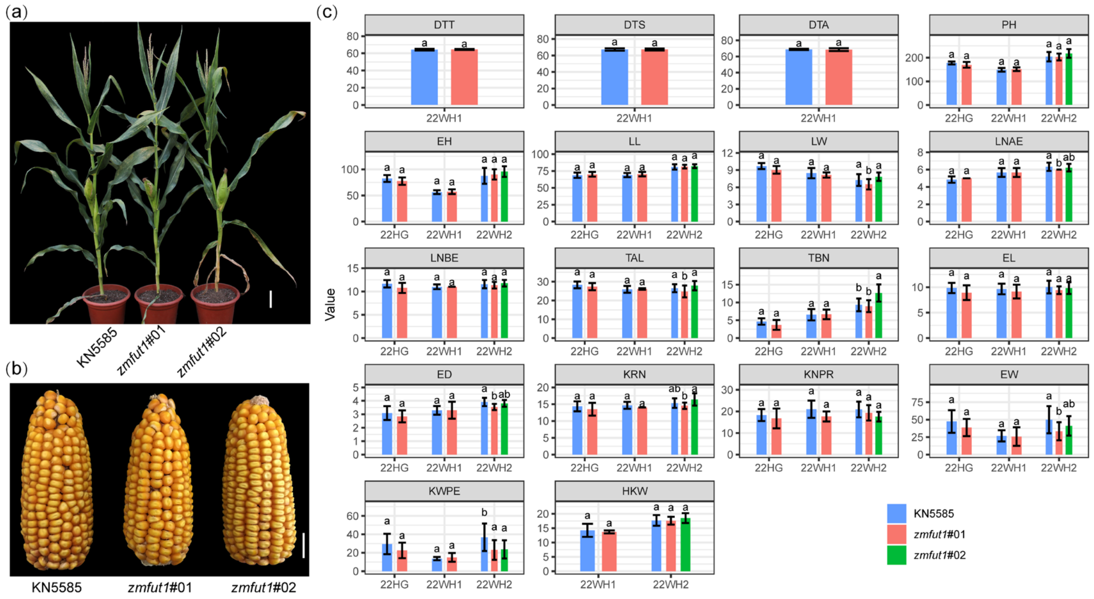

Figure S16 The agronomic traits of *zmfut1*-knockout lines.

The plant (a) and ear performance (b) of KN5585 and *zmfut1*-knockout lines. Scale bars in (a) and (b), 10 cm for plants, 2 cm for ears. (c) The agronomic traits of KN5585 and *zmfut1*-knockout lines in three environments (22HG, 22WH1, 22WH2). DTT, days to tasseling; DTS, days to silking; DTA, days to anthesis; PH, plant height (cm); EH, ear height (cm); LL, ear leaf length (cm); LW, ear leaf width (cm); LNAE, leaf number above ear; LNBE, leaf number below ear; TAL, tassel main axis length (cm); TBN, tassel branch number; EL, ear length (cm); ED, ear diameter (cm); KRN, kernel row number; KNPR, kernel number per row; EW, ear weight (g); KWPE, kernel weight per ear (g); HKW, hundred kernel weight (g). Difference letters indicate significant difference at fdr ≤ 0.05 level via Fisher's LSD test.

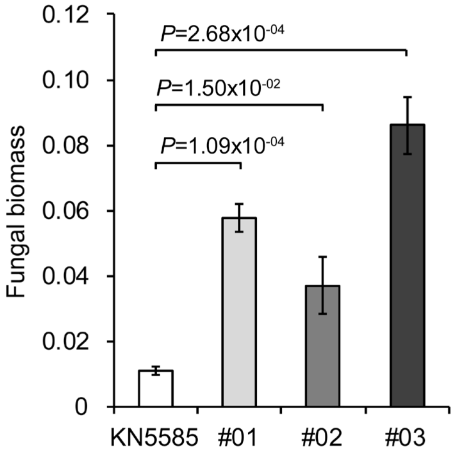

Figure S17 The fungal biomass of wildtype and *mybr92*-knockout lines inoculated with *C. heterostrophus* pathogen.

Values are means ± SD. Differences among groups were analyzed by Student's *t*-test.

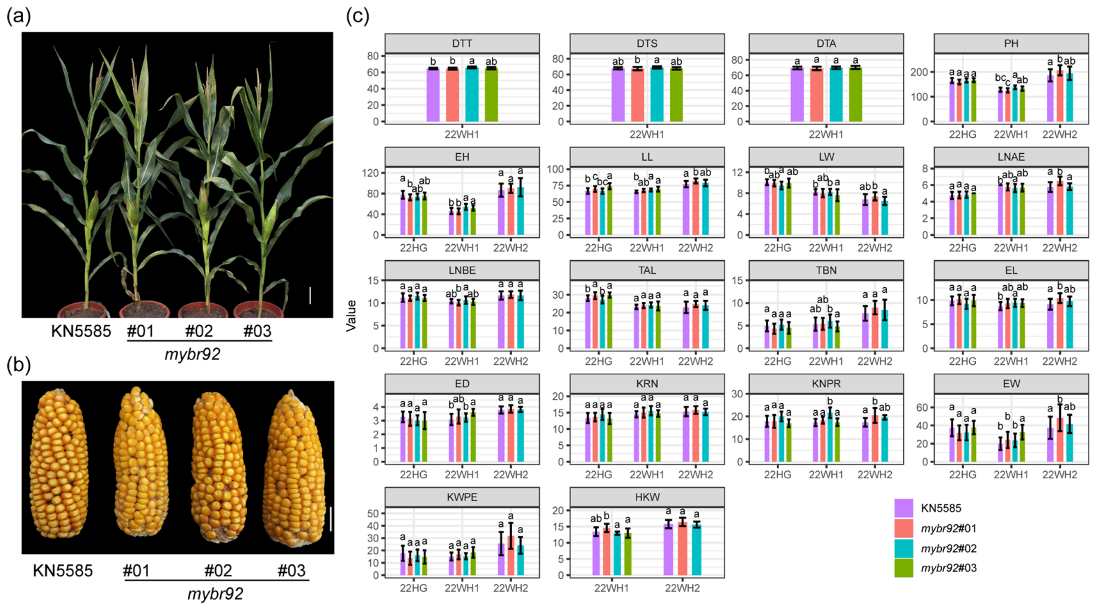

Figure S18 The agronomic traits of *mybr92*-knockout lines.

The plant (a) and ear performance (b) of KN5585 and *mybr92*-knockout lines. Scale bars in (a) and (b), 10 cm for plants, 2 cm for ears. (c) The agronomic traits of KN5585 and *mybr92*-knockout lines in three environments (22HG, 22WH1, 22WH2). DTT, days to tasseling; DTS, days to silking; DTA, days to anthesis; PH, plant height (cm); EH, ear height (cm); LL, ear leaf length (cm); LW, ear leaf width (cm); LNAE, leaf number above ear; LNBE, leaf number below ear; TAL, tassel main axis length (cm); TBN, tassel branch number; EL, ear length (cm); ED, ear diameter (cm); KRN, kernel row number; KNPR, kernel number per row; EW, ear weight (g); KWPE, kernel weight per ear (g); HKW, hundred kernel weight (g). Difference letters indicate significant difference at fdr ≤ 0.05 level via Fisher's LSD test.

(a)

ZmFUT1	InDel -1485	InDel 1240	SNP 3907
ZmFUT1a	6bp del	-	-
ZmFUT1b	-	0	-
ZmFUT1c	-	-	G

MYBR92	SNP 463	SNP 6398
MYBR92a	T	-
MYBR92b	-	G

(b)

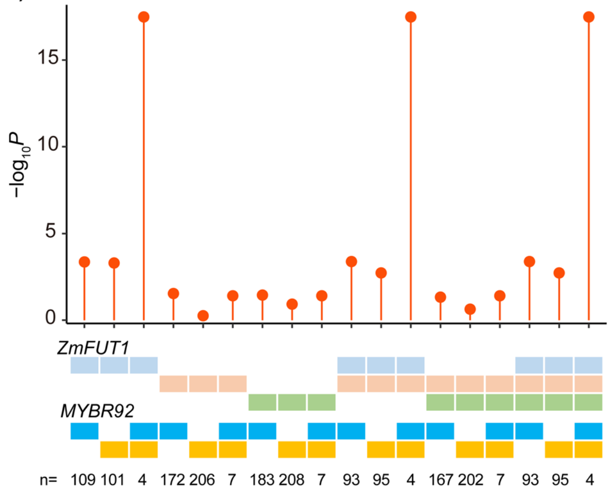

Figure S19 Haplotype-based association between *ZmFUT1* and *MYBR92*.

(a) The resistant haplotypes of five polymorphism sites. (b) Haplotype-based association between *ZmFUT1* and *MYBR92* at five polymorphism sites. The number of lines carrying resistant haplotypes was represented below.
